# Global distribution of malaria-resistant MHC-HLA alleles: the number and frequencies of alleles and malaria risk

**DOI:** 10.1186/1475-2875-13-349

**Published:** 2014-09-03

**Authors:** László Zsolt Garamszegi

**Affiliations:** Department of Evolutionary Ecology, Estación Biológica de Doñana-CSIC, c/Americo Vespucio, s/n, 41092 Sevilla, Spain

**Keywords:** Host-parasite interaction, Major histocompatibility complex, Malaria, Parasite transmission, Vector-borne infectious diseases

## Abstract

**Background:**

The major histocompatibility complex (MHC) is the most polymorphic genetic region in vertebrates, but the origin of such genetic diversity remains unresolved. Several studies have demonstrated at the *within*-population level that individuals harbouring particular alleles can be less or more susceptible to malaria, but these do not allow strong generalization.

**Methods:**

Here worldwide data on the frequencies of several hundred MHC alleles of the human leucocyte antigen (HLA) system in relation to malaria risk at the *between*-population level were analysed in a phylogenetic framework, and results for different alleles were quantitatively summarized in a meta-analysis.

**Results:**

There was an overall positive relationship between malaria pressure and the frequency of several HLA alleles indicating that allele frequencies increase in countries with strong malaria pressure. Nevertheless, considerable heterogeneity was observed across alleles, and some alleles showed a remarkable negative relationship with malaria risk. When heterogeneities were partitioned into different organization groups of the MHC, the strongest positive relationships were detected for alleles of the HLA-A and HLA-B loci, but there were also differences between MHC supertypes that constitute functionally distinct nucleotide sequences. Finally, the number of MHC alleles that are maintained within countries was also related to malaria risk.

**Conclusion:**

Therefore, malaria represents a key selection pressure for the human MHC and has left clear evolutionary footprints on both the frequencies and the number of alleles observed in different countries.

**Electronic supplementary material:**

The online version of this article (doi:10.1186/1475-2875-13-349) contains supplementary material, which is available to authorized users.

## Background

Malaria, caused by the members of the genus *Plasmodium*, is one of the worst scourges of both mankind and wildlife representing a research agenda with global concerns for human health and conservation [1, 2]. Several thousand parasite strains and species with a broad spectrum of host exploitation strategies exist that infect hundreds of different hosts from the major terrestrial vertebrate taxa [3, 4] with a considerable variation among populations in disease susceptibility and mortality rates [5–7]. Such variance in both parasite life history and host’s resistance is a net effect of several climatic, biological and socio-ecological factors. From the evolutionary viewpoint, observed interspecific or interstrain variation in virulence is the result of a long-lasting, co-evolutionary battle between the parasites and their hosts that is played at the molecular level. One of the signatures of such an interaction between parties at the phylogeographic scale is the relationship between malaria pressure and immunogenetic factors that determine disease susceptibility. It is a well known phenomenon, for example, that in countries suffering from high mortality and morbidity costs of malaria, the frequency of alleles, such as those coding for sickle-cell anaemia or beta thalassaemia, that also confer protection against the escalation of pathological symptoms caused by the infection is also higher than in malaria free areas [8, 9]. Similarly, such a mechanism can also be manifested for factors that incur higher vulnerability to malaria with decreasing frequencies of alleles under high parasite burden.

The major histocompatibility complex (MHC) in humans and in other vertebrate taxa harbours several genes that by coding important molecules for antigen recognition and presentation are crucial for the efficient functioning of the immune system against intra- and extracellular parasites, including the malaria agents [10, 11]. MHC-mediated resistance to malaria can be achieved via two major functional pathways. The antigen presentation of MHC class I proteins, by triggering cytotoxic T cells against intracellular parasites, may play an important role during the liver-stage infection, while class II molecules can mediate the clearance of parasitized erythrocytes from the bloodstream through the stimulation of helper T cells [12]. The mechanistic link between malaria and the MHC seems evident from some population studies of humans and birds, in which the between-individual variation in the prevalence of certain alleles was associated with tolerance or susceptibility of infection [12–18]. However, such patterns observed within few populations are hard to translate into evolutionary mechanisms that generate variations in malaria risk between countries. Several thousand MHC alleles have been described in humans worldwide [19], but population-specific links for a handful of them have limited value for making generalizations about the co-evolution of malaria and MHC-mediated immunity. The existence of the uniquely large genetic variation at the MHC still begs for a universal answer.

The maintenance of an extreme genetic diversity at the MHC, manifested as a large number of alleles and by the differences in nucleotide sequence among alleles, is one of the most challenging evolutionary puzzles [20–23]. Most hypotheses aiming at explaining this diversity focus on pathogen-mediated balancing selection, emphasizing that MHC polymorphism evolves under the pressure of a diverse parasite fauna. A straightforward prediction of the hypothesis about pathogen-mediated balancing selection is that between-population variation in MHC diversity should positively correlate with between-population variation in parasite richness [24]. This relationship is expected, because individuals with higher MHC polymorphism should have a wider range of genes that offer protection against a higher number of pathogens. In addition, such a mechanism may be in effect at a higher level, as populations maintaining a higher number of functioning alleles through the diversity of individual genotypes could survive longer under malaria burden than populations with less polymorphism.

To test if increased malaria pressure selects for the increased frequency and/or number of the resistance alleles within populations the evolutionary link between malaria and the population genetics of the MHC in humans was studied. Global patterns of MHC allele frequencies were investigated in relation to country-level estimates of malaria risk focusing on a large set of human leukocyte antigens (HLA). Considering a co-evolutionary process [25, 26], if increased parasitism favours efficient immune responses in the host, components that enhance the resistance to infection will accumulate in areas with a strong malaria pressure, while susceptibility factors that make individuals prone to the disease will be removed over evolutionary time scales. Such causal mechanisms will result in positive relationships at the above-individual level (i.e., across different populations of hosts) between resistance traits and disease risk, while negative relationships for resistance traits. Testing for correlations between allele frequencies and malaria risk for a large number of alleles will result in significant associations in 5%, simply by chance. However, if the detected correlations are shaped by biological mechanisms, one should expect to find different patterns among groups of alleles with different functions.

## Methods

### Malaria risk

Data for malaria risk originated from the WHO Malaria Report, which collects the yearly results of the worldwide monitoring programme [5]. In that Report, local malaria risk for each endemic country was calculated as the number of people inhabiting low- and high-transmission areas relative to the total population size. Such an estimate strongly correlates with other variables that also reflect the impact of malaria (e.g., death rate: r = 0.582, P <0.001, N = 81; proportion of inpatient cases: r = 0.591, P <0.001, N = 65; proportion of confirmed cases: r = 0.543, P <0.001, N = 104). Importantly, the above source of data provides information on the conjoint effect of different *Plasmodium* species. However, given that the estimated risk of malaria from such combined data strongly correlates with death rate (as shown above), one can infer that the focal variable mostly reflects the pressure by the most virulent parasite, the *P. falciparum*. Due to efficient prevention efforts and other, different, anthropogenic effects as well as climate change, malaria risk may show a recent increase or decrease in some countries. However, such variation seems negligible when compared to the between-country variation: the number of malaria cases shows high repeatability within countries (R = 0.893, P <0.001, N = 106 countries), based on 20 years of data. Moreover, the use of the proportion of the population that is exposed to transmission as an estimate of malaria risk involves less sensitivity to yearly fluctuations caused by prevention programmes and global warming than the use of an estimate based on the number of observed cases [1]. Therefore, risk estimates based on the 2011 Report were used and it was assumed that such data reflect the country-specific malaria burden. Global malaria risk was assessed for all countries for which data on MHC frequency were available. The impact of malaria was scored along a three-state scale: no impact, weak impact, strong impact. Countries that are not listed by the WHO as endemic countries were considered to have zero (0) risk of malaria. Endemic countries without considerable death toll (<100 death cases in the last 20 years) were considered in a moderate state of global risk (1), while endemic countries with large numbers of death cases (>100 death cases in the last 20 years) were regarded as representative of strong malaria pressure (2). Intermediate states between these scores are biologically meaningful, thus the estimate of global risk was treated along a continuous scale.

### MHC allele frequency

Data were extracted from the Allele Frequency Net Database, which is a central source for different polymorphic areas in the Human Genome Project [19]. MHC-HLA allele frequencies are tabulated at the population level, with more than one estimate in each country. Based on such multiple entries, the within-country repeatability of frequencies of alleles that were screened in at least ten countries and two localities was calculated. Repeatability estimates averaged across different alleles indicated that, in general, there was a consistent variation in allele frequencies at the within-country level (R ± standard error (s.e.) = 0.443 ± 0.012 for 229 alleles, of which 202 were significant). Therefore, by weighting each population-specific datum according to the associated sample size (number of people tested), country-specific means were calculated and it was assumed that such estimates are biologically reliable. Note that certain differences among populations could be independent of sample size, e.g. some populations may share some alleles. The potentially confounding effect of such genetic similarity between populations is considered, at least partially, in the phylogenetic analyses (see below).

MHC alleles were categorized into loci as in the original source following the standards [27]. Alleles were also sorted into different supertypes, which separate groups of MHC molecules based on their peptide-binding specificity, as indicated by the specific amino-acid sequence motifs [28, 29]. Grouping of MHC molecules into supertypes based on their peptide-binding repertoire was originally suggested to be useful for developing epitope-based vaccines [30]. However, such clustering may be straightforward in the evolutionary context, as peptide-binding specificity should serve as a functional unit for selection as excreted by different parasitic antigens.

### Phylogenetic comparative approaches

Country-based estimates of the focal variables are statistically non-independent observations, because countries separated by short distance may show more similarities than countries located at larger distances. Closely situated countries may share several factors (e.g., climate, vector populations, malaria strains, genetically inherited resistance) that determine patterns of both malaria burden and MHC population genetics and can raise spurious correlations between them. This problem of statistical non-independence can be effectively handled by phylogenetic comparative methods when similarities between entities are considered along a tree structure [31]. Unfortunately, detailed phylogenetic information that separates human populations at the country level and that depict the phylogeography of different *Plasmodium falciparum* strains at the global scale are missing. Therefore, to statistically control for the non-independence of data due to common descent a ‘phylogenetic tree’ based on the geographic distances between countries was used assuming that such distances reflects genetic distances of both the hosts and the parasites [32, 33]. Based on the geographic coordinates of the sampling localities, a distance matrix was created, which was then used for clustering by Unweighted Pair Group Method with Arithmetic Mean (UPGMA) [34] to derive a tree to describe similarities between countries based on their physical distance (Additional file 1). This phylogenetic tree was incorporated in a comparative framework [35] to test for the association between country-specific means of allele frequency and malaria risk while controlling for similarities between countries that arise from their physical distance. Specifically, phylogenetic generalized least square (PGLS) methods were used to account for the expected similarity in phenotypes as described by the variance-covariance matrix as defined by the hierarchical association structure of the data [36]. As this matrix is calculated based on the distances (instead of true phylogenetic distances) between countries, the approach is formally equivalent to a spatial autocorrelation model.

For each allele, if the corresponding sample size was larger than five (i.e., data on both allele frequency and malaria risk were available for at least five countries), two models were built: one with malaria risk as a continuous (risk at local scale) and another with risk as a discrete (risk at global scale) predictor, both with allele frequency as a response variable. From these models, by using information on the corresponding *t* values of the estimated slope parameters and the residual degrees of freedom, the correlation between the focal traits was calculated in the form of the ‘r’ (Pearson correlation coefficient) effect size [37, 38]. Note that this study does not compare the significance of particular effects (i.e. whether the relationship between the frequency of a particular allele correlates significantly with malaria risk), because it is meaningless when sample sizes vary between tests. Instead, by using a meta-analytic approach (see below), it focuses on the magnitude of these effects and the precision by which these can be estimated. Consequently, no correction for multiple testing was required (which would apply to P values).

### Meta-analyses

The above analyses provided several hundred correlations. To statistically summarize results over the entire sample of alleles examined, meta-analyses were performed. By doing so, each particular relationship was weighted by its sample size (number of countries) to emphasize particular effects proportionally based on the precision by which they can be measured [39]. The analyses relied on the normalized score of r, Fisher’s Z, and on random-effect models that assume considerable variability in the effect sizes across alleles to deal with their potentially different evolutionary role. To test for such potential variation in effect size, tests of heterogeneity that quantitatively estimated the difference in the strength of correlation corresponding to different alleles were carried out. To examine if the organization of alleles within the MHC and their potentially different functions were responsible for the heterogeneity of correlations, the effect of MHC loci as a moderator variable was examined by partitioning heterogeneities across the major organizational groups. All analyses were performed in the R statistical environment [40] after the appropriate transformation of variables.

## Results

### Relationships between malaria risk and the frequency of particular alleles

**Figure 1:**
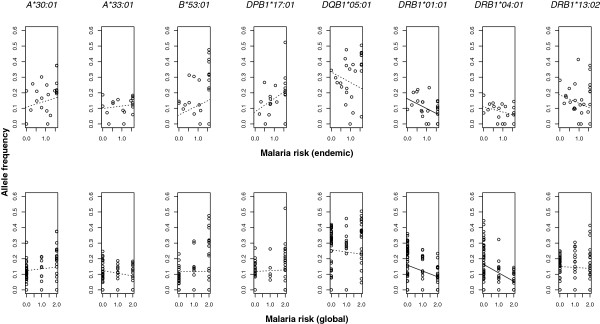
**The across-country relationship between malaria risk and the allele frequency of MHC alleles were previously shown to be involved in resistance or susceptibility to malaria in within-population studies **[12–17]**.** Upper panels show the relationships for local estimates of malaria risk in malaria endemic countries. Lower panels are for global estimates of malaria risk for all countries for which data on allele frequency were available. Resistance factors: B*53:01, DQB1*05:01, DRB1*01:01, and DRB1*13:02; Susceptibility factors: A*30:01, A*33:01, DPB1*17:01, and DRB1*04:01. Points are country-specific estimates and lines are from regressions that account for the spatially non-random association of countries (dashed: non-significant, solid: significant).

Figure 1 illustrates the focal relationship for some of those alleles that emerged as potential resistance or susceptibility factors in within-population studies and that can serve as external controls for the higher-level approaches developed here. Comparing patterns that were previously observed *within* countries with patterns that can be observed *between* countries suggests that correlations at the between-country level can also provide meaningful results. Some of these (e.g., for HLA-DRB1*01:01 or HLA-DRB1*04:01) supported the hypothesis that malaria risk varies in parallel with MHC allele frequency across countries.

Focusing on the entire sample as a whole (Additional file 2) it was found that 86 (15%) of the 585 alleles showed a significant association with local malaria risk, which is statistically more evidence than could emerge by chance (*χ*2 = 115.90, P <0.001). The occurrences of positive and negative relationships were not symmetric, as 373 were positive and 212 were negative (*χ*2 = 44.31, P <0.001). The parallel results for global malaria risk showed a similar picture. Out of 891 correlations, 83 (9%) were significant (*χ*2 = 34.93, P <0.001), and the ratio of positive and negative relationships was 547/344 (*χ*2 = 46.25, P <0.001).

### Malaria risk and allele frequency: general patterns emerging in a meta-analysis

**Figure 2:**
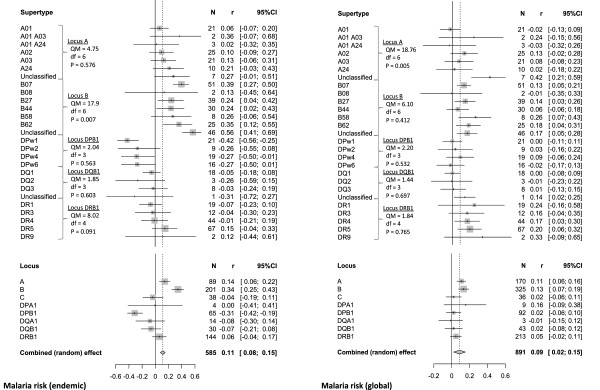
**Meta-analysis summary of the relationship between malaria risk and allele frequency.** Local malaria risk (only endemic countries) is shown on the left and global estimates of risk (all countries) on the right. On the top, effect sizes are grouped into supertypes. Statistical results demonstrate whether supertype-specific effect sizes are significantly different within a particular locus. Comparisons of effect sizes across loci are shown on the bottom. Squares and horizontal lines show mean effect sizes (r) and the associated 95% confidence intervals (95% CI), respectively. Diamonds summarize the entire data (vertical endpoints: overall mean effect size, horizontal endpoints: 95% CI). N is the number of alleles.

**Figure 3:**
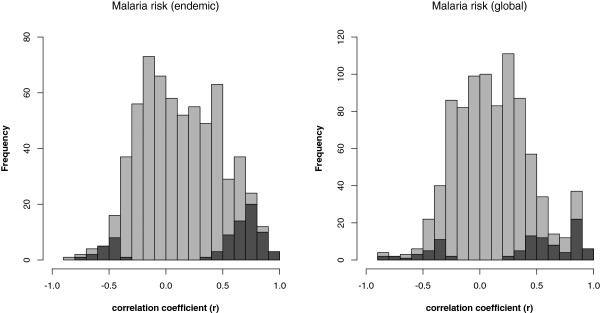
**Distribution of effect sizes for the relationship between malaria risk and allele frequency for different MHC alleles.** Left panel: the distribution of detected correlations when only malaria-endemic countries were considered. Right panel: the distribution of correlations for global malaria. Dark grey bars cover correlations that were statistically significant at the P <0.05 level.

A statistical summary over the entire sample was obtained by balancing for differences in data availability across alleles in a meta-analysis (Figure 2). The overall effect size was significantly larger than zero, suggesting a general tendency for a positive relationship (random-effect models, local malaria risk: r = 0.114, 95% CI: 0.078/0.150, P <0.001, N = 585; global malaria risk: r = 0.080, 95% CI: 0.060/0.100, P <0.001, N = 891). However, tests of heterogeneity showed high variability among alleles, implying that correlations for particular alleles may correspond to different biological mechanisms (local malaria risk: Q = 1,062.82, P <0.001, I^2^ = 44.92%; global malaria risk: Q = 1,456.31, P <0.001, I^2^ = 38.45%). Therefore, although there is general evidence for a positive relationship between MHC allele frequency and malaria risk, this is not an exclusive rule, as some alleles exhibit a decline in frequency in relation to increasing *Plasmodium* parasitism, and the strength of relationship may vary from alleles to alleles (see Figure 3 and Additional file 2).

A partitioning of effect sizes among different HLA supertypes and loci suggested that some of the heterogeneity could be attributed to the organization of alleles (Figure 2). HLA-A and HLA-B Class I alleles showed systematically more positive effects than Class II loci, suggesting that different evolutionary mechanisms may apply to alleles that mediate immunity to malaria during the liver- and blood-stages of infection. Moreover, there were also differences between particular MHC supertypes (Figure 2).

### Malaria risk and the number of HLA alleles maintained in populations

**Figure 4:**
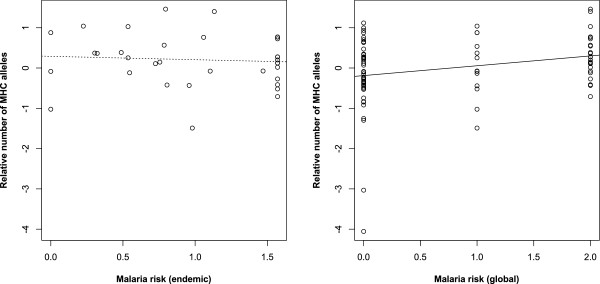
**The relationship between malaria risk and the number of MHC alleles present in different countries.** The relationship for malaria-endemic countries, local estimates of malaria risk, is shown on the left. The right panel includes every country considered on the global scale, when malaria risk is treated along the no impact – weak impact – strong impact axis. Points are country-specific estimates and lines are from regressions that account for the spatially non-random association of countries (dashed: non-significant, solid: significant).

Increased malaria pressure should generate allelic richness if several alleles offer protection against the disease. Based on alleles that have non-zero frequency locally, the number of alleles that were detected in each country was calculated and it was tested if malaria risk is related to the number of different MHC alleles that are maintained in human populations. A statistical control for differences in sampling (as MHC allelic diversity was positively related to the number of individuals screened: β ± s.e. = 0.349 ± 0.061, P <0.001), showed that the global malaria risk was a significant predictor of MHC polymorphisms (local malaria risk: β ± s.e. = −0.120 ± 0.180, P = 0.507; global malaria risk: β ± s.e. = 0.205 ± 0.100, P = 0.043, Figure 4).

## Discussion

The current analysis targeted global patterns of variation of the MHC for several hundred alleles, thus the general results observed here have wide evolutionary implications for MHC polymorphism. The results imply that even though several parasites can be linked to particular MHC alleles, malaria-mediated selection per se can impose detectable evolutionary forces for the population genetics of the MHC. These mechanisms extend to a large part of the MHC molecule, since several alleles within entire organizational units at the higher level (loci, supertypes) can accumulate in malaria-infected areas. Hence, malaria seems to be responsible for generating a considerable amount of the MHC polymorphism in humans.

One of the key findings of the study was that the frequency of a considerable number of alleles was positively related to the risk of malaria with magnitude that fall within the wide range from small to large effect sizes, while for a good number of alleles negative relationships were observed. Moreover, not only the frequencies of particular alleles were affected, but also a wider MHC allele spectrum (i.e., the number of alleles maintained within a population) was observed in association with increased malaria pressure (at least when malaria risk was regarded at a global scale, i.e. when focusing on the contrast between endemic and non-endemic countries). These patterns are, in general, in line with the hypothesis that in malaria-endemic areas, the accelerated arms race between hosts and parasites enhances: i) the accumulation of the malaria resistant MHC alleles and, ii) the maintenance of a broad spectrum of MHC alleles in the host population if malaria is permanently present. These effects, acting at two different levels, may offer efficient protection against the quickly evolving strains of the parasite. The study, hence, provides strong support for that malaria-mediated balancing selection is operating on the HLA gene complex.

Interpretations about causal mechanisms are valid if the observed correlations reflect the evolutionary state in which the parasite is one step ahead and it represents an important selection factor for host immunity, similarly to well-known genetic adaptations to malaria through sickle cell or thalassaemia genes [41]. However, when considering a co-evolutionary arms race, the opposite causal mechanism may also be at work, if one assumes that patterns of the outcomes of malaria infection reflect the parasite’s response to host defense and not vice versa. Such an alternative explanation is unlikely, because both local and global estimates of malaria risk provided meaningful correlations, which contradicts the causal scenario about MHC allele frequencies influencing the spread of parasites. This contradiction arises because the worldwide distribution of *Plasmodium* is constrained by several factors (e.g., climate, vector abundance, socio-economic factors), and it is improbable that differences in host immunity alone can establish detectable differences when endemic and malaria-free countries are compared. Even if host resistance constrains parasite distribution to some degree, such effects would have remained blurred at worldwide level due to several uncontrolled factors. As a result, host effects on parasites should only appear within the malaria-endemic region. Taken together, association between MHC allele frequency and malaria risk on a broad scale is more likely to mirror the causal mechanism, in which malaria is in a leading role and constitutes a selection pressure for host immunity.

Patterns at the within-country context do not necessarily translate into patterns at the between-country context. Previous studies, by comparing the consequence of malaria infection among individuals harbouring different alleles in certain countries, successfully identified some resistance and susceptibility factors [12–17]. However, these findings were not broadly generalizable, and the relationships remained applicable to the specific locality where the data came from. For example, in a Gambian population, DRB1*13:02 and B*53:01 were associated with reduced susceptibility to severe malaria [12], while similar resistance roles were not transparent within the Dogon ethnic group in Mali [14]. Such a geographical variation in the magnitude and the direction of the relationship between the presence of a certain MHC allele and the degree of protection it involves against malaria was also demonstrated in a bird species [42]. Data from 13 populations of the house sparrow (*Passer domesticus*) suggests a role for diversifying selection that favours different host allelic lineages in different populations. Similar mechanisms may explain why one could not necessarily reproduce within-population relationships at a higher level, when different countries are compared. Hence, different co-evolutionary mechanisms may operate at different levels.

The strength of the relationship between allele frequency and malaria risk was heterogeneous across HLA class, loci and supertypes. In general, alleles belonging to the HLA A and B groups could generally be characterized by stronger positive associations than other HLA groups. This observation is in good agreement with previous studies, suggesting that HLA B could be under a higher selective pressure from pathogens [24, 43, 44]. The fact that MHC class I molecules have the strongest positive effects indicates that antigen presentation during the liver-stage infection plays an important role in malaria defence, and the corresponding genetic machinery is thus under strong selection. Interestingly, frequencies of alleles of the DPB locus showed a generally strong negative association with malaria risk (but only when the endemic countries were compared). HLA-DPB belongs to the HLA class II and plays a central role in the immune system by presenting peptides originating from extracellular proteins [45–47]. It seems that for some reason such functions have negative consequences for malaria resistance, thus the frequency of these alleles declines in areas where malaria has a strong mortality cost.

Given that observed patterns of prevalence and virulence are the products of an evolutionary race between hosts and parasites, defence programmes are now to begin embracing a phylogenetic perspective that helps understand the evolution of drug resistance and predicts the success of vector control [48, 49]. Accordingly, the results presented here may contribute to the global fight against malaria. For example, the success or failure of malaria campaigns in different countries can be re-interpreted in light of the frequency of different MHC alleles in the populations. Knowledge about the occurrence of susceptibility factors may also help channel investments to countries according to their immunological sensitivity. Furthermore, discriminating between MHC supertype groups is relevant for developing epitope-based vaccines; thus, comparing nucleotide sequences of alleles that demonstrate similar relationships with malaria risk may help identify the molecular basis of resistance and susceptibility, contributing to drug development.

## Conclusions

This study, focusing on the distribution of several hundred alleles by integrating comprehensive resources at a worldwide level, investigates how host-parasite interactions occurring at the molecular level can affect the population genetics of the human MHC in relation to malaria. Although previous studies have also targeted similar issues at the within-population level, none of these provided strong and generalizable evidence for parasite virulence affecting the evolution of host immunogenetics. The current work, on the other hand, provides novel insights, because it powerfully demonstrates, which group of MHC alleles (supertypes of the HLA-B) accumulates under strong malaria pressure. The results also offer an explanation for the uniquely high genetic polymorphism of the MHC, which is one of the most challenging mysteries in evolutionary biology. Finally, the findings may have relevance for defence programs, as the alleles identified as having possible evolutionary roles may help elucidate the molecular background of malaria resistance.

## Electronic supplementary material

Additional file 1: **The “phylogenetic” tree of countries based on their geographic distance.** Description: The “phylogenetic tree” that was incorporated in a comparative framework to test for the association between country-specific means of allele frequency and malaria risk while controlling for similarities between countries that arise from their physical distance. (PDF 228 KB)

Additional file 2: **The association between the frequency of particular MHC alleles and malaria risk at the local and global scales.** Description: Results from phylogenetic analyses that consider the statistical non-independence of data as summarized in Additional file 1. (DOC 1 MB)
