# Supplementary figures and images for: Global distribution of malaria-resistant MHC-HLA alleles: the number and frequencies of alleles and malaria risk
Source: Malar J. 2014 Sep 3;13:349. doi: 10.1186/1475-2875-13-349 (PMC4162943; doi:10.1186/1475-2875-13-349)

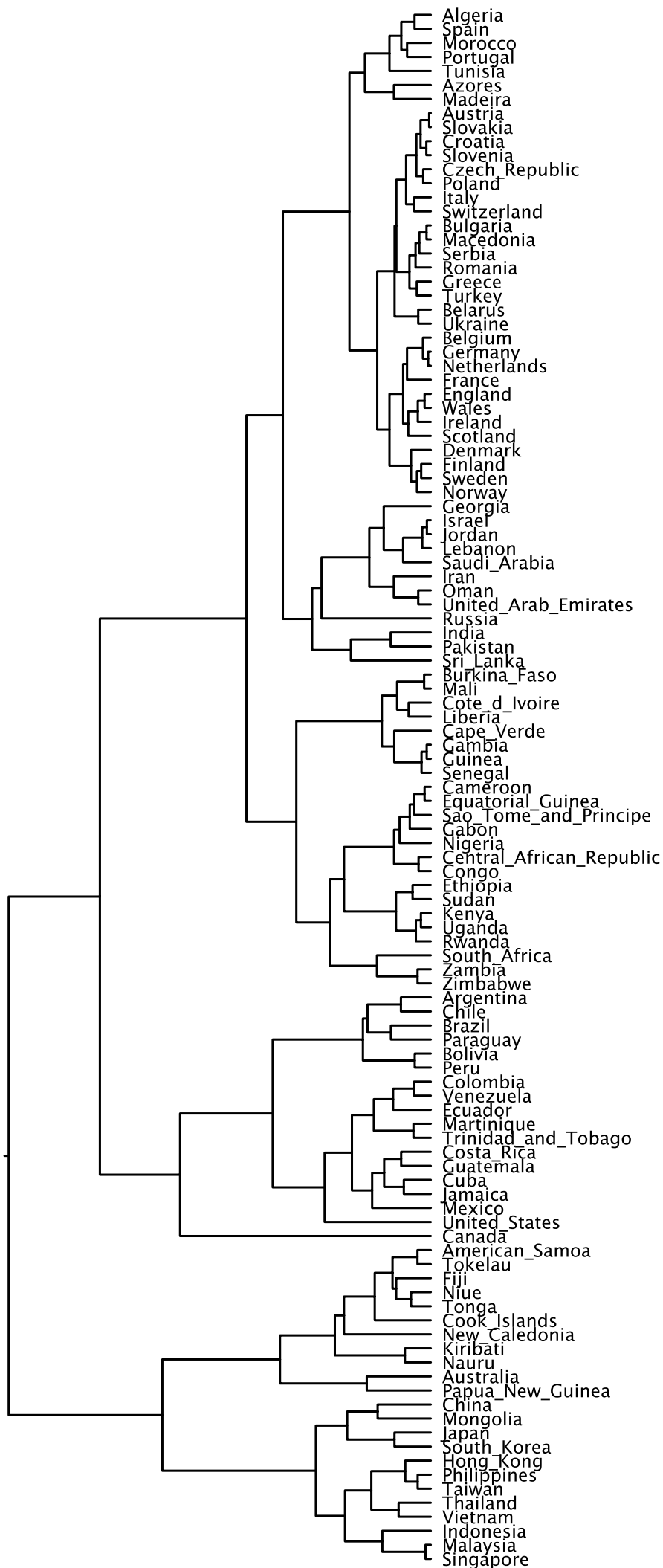

Supplement: Supplementary file 1 — Additional file 1: The “phylogenetic” tree of countries based on their geographic distance. Description: The “phylogenetic tree” that was incorporated in a comparative framework to test for the association between country-specific means of allele frequency and malaria risk while controlling for similarities between countries that arise from their physical distance. (PDF 228 KB) [file 12936_2014_3382_MOESM1_ESM.pdf]
