# Supplementary material for: Global distribution of malaria-resistant MHC-HLA alleles: the number and frequencies of alleles and malaria risk
Source: Malar J. 2014 Sep 3;13:349. doi: 10.1186/1475-2875-13-349 (PMC4162943; doi:10.1186/1475-2875-13-349)
Supplement: Supplementary file 2 — Additional file 2: The association between the frequency of particular MHC alleles and malaria risk at the local and global scales. Description: Results from phylogenetic analyses that consider the statistical non-independence of data as summarized in Additional file 1. (DOC 1 MB) [file 12936_2014_3382_MOESM2_ESM.doc]

**The association between the frequency of particular MHC alleles and malaria risk at the local and global scales.** Results from phylogenetic analyses that consider the statistical non-independence of data as summarized on Supplementary Figure 1.

| **Allele** | **Local malaria risk** | | | **Global malaria risk** | | |
| --- | --- | --- | --- | --- | --- | --- |
|  | *N* | *r* | *P* | *N* | *r* | *P* |
| *A*01:01* | 23 | -0.138 | 0.531 | 54 | -0.244 | 0.076 |
| *A*01:02* | 18 | 0.315 | 0.203 | 34 | 0.309 | 0.076 |
| *A*01:03* | 8 | 0.238 | 0.570 | 21 | -0.207 | 0.369 |
| *A*01:06* | 5 | 0.769 | 0.129 | 12 | 0.189 | 0.556 |
| *A*01:09* |  |  |  | 5 | 0.559 | 0.327 |
| *A*02:01* | 27 | -0.320 | 0.104 | 64 | -0.146 | 0.249 |
| *A*02:02* | 23 | 0.385 | 0.070 | 48 | 0.266 | 0.067 |
| *A*02:03* | 13 | 0.308 | 0.305 | 35 | 0.096 | 0.582 |
| *A*02:04* | 15 | -0.140 | 0.618 | 34 | 0.251 | 0.152 |
| *A*02:05* | 23 | 0.023 | 0.916 | 54 | 0.070 | 0.615 |
| *A*02:06* | 19 | 0.050 | 0.838 | 49 | -0.119 | 0.417 |
| *A*02:07* | 12 | 0.147 | 0.648 | 33 | -0.162 | 0.369 |
| *A*02:08* | 10 | 0.175 | 0.629 | 28 | 0.042 | 0.830 |
| *A*02:09* | 9 | 0.802 | 0.009 | 23 | 0.320 | 0.137 |
| *A*02:10* | 9 | -0.050 | 0.899 | 25 | 0.032 | 0.881 |
| *A*02:11* | 18 | -0.017 | 0.948 | 37 | 0.351 | 0.033 |
| *A*02:12* | 11 | 0.181 | 0.595 | 27 | 0.331 | 0.092 |
| *A*02:13* | 11 | 0.203 | 0.549 | 25 | 0.175 | 0.404 |
| *A*02:14* | 15 | 0.463 | 0.082 | 30 | -0.120 | 0.527 |
| *A*02:16* | 10 | 0.366 | 0.298 | 26 | 0.349 | 0.080 |
| *A*02:17* | 19 | -0.049 | 0.842 | 37 | 0.068 | 0.687 |
| *A*02:18* |  |  |  | 22 | -0.192 | 0.391 |
| *A*02:19* | 10 | -0.127 | 0.727 | 25 | -0.174 | 0.404 |
| *A*02:20* | 11 | 0.639 | 0.034 | 25 | 0.398 | 0.049 |
| *A*02:21* | 8 | -0.043 | 0.920 | 20 | 0.284 | 0.225 |
| *A*02:22* | 11 | 0.082 | 0.810 | 29 | 0.457 | 0.013 |
| *A*02:24* | 11 | 0.217 | 0.522 | 26 | 0.171 | 0.404 |
| *A*02:25* | 13 | 0.117 | 0.704 | 27 | -0.008 | 0.969 |
| *A*02:26* | 10 | 0.366 | 0.299 | 23 | 0.051 | 0.816 |
| *A*02:27* |  |  |  | 12 | 0.457 | 0.135 |
| *A*02:28* |  |  |  | 9 | -0.491 | 0.179 |
| *A*02:29* |  |  |  | 11 | -0.261 | 0.438 |
| *A*02:30* |  |  |  | 9 | 0.512 | 0.158 |
| *A*02:33* |  |  |  | 9 | -0.289 | 0.451 |
| *A*02:34* |  |  |  | 10 | 0.557 | 0.094 |
| *A*02:35* |  |  |  | 10 | -0.373 | 0.289 |
| *A*02:36* | 6 | 0.191 | 0.717 | 15 | 0.257 | 0.355 |
| *A*02:37* |  |  |  | 8 | 0.570 | 0.140 |
| *A*02:38* |  |  |  | 8 | 0.570 | 0.140 |
| *A*02:40* |  |  |  | 10 | 0.227 | 0.527 |
| *A*02:42* |  |  |  | 6 | 0.679 | 0.138 |
| *A*02:44* |  |  |  | 7 | -0.305 | 0.506 |
| *A*02:45* |  |  |  | 7 | -0.128 | 0.785 |
| *A*02:46* |  |  |  | 8 | -0.855 | 0.007 |
| *A*02:49* |  |  |  | 7 | 0.801 | 0.031 |
| *A*02:51* |  |  |  | 6 | 0.410 | 0.420 |
| *A*02:54* |  |  |  | 5 | 0.875 | 0.052 |
| *A*02:55* |  |  |  | 5 | 0.875 | 0.052 |
| *A*02:57* |  |  |  | 6 | 0.200 | 0.704 |
| *A*02:58* |  |  |  | 5 | 0.401 | 0.503 |
| *A*02:60* |  |  |  | 5 | -0.404 | 0.500 |
| *A*03:01* | 21 | 0.169 | 0.464 | 53 | -0.181 | 0.194 |
| *A*03:02* | 15 | -0.195 | 0.487 | 43 | 0.087 | 0.581 |
| *A*03:05* |  |  |  | 10 | 0.334 | 0.346 |
| *A*03:07* |  |  |  | 6 | 0.800 | 0.056 |
| *A*03:08* |  |  |  | 8 | 0.586 | 0.127 |
| *A*03:10* |  |  |  | 5 | 0.875 | 0.052 |
| *A*11:01* | 18 | -0.141 | 0.576 | 54 | -0.125 | 0.369 |
| *A*11:02* | 11 | 0.233 | 0.491 | 25 | 0.077 | 0.714 |
| *A*11:03* | 8 | 0.182 | 0.667 | 20 | 0.375 | 0.103 |
| *A*11:04* | 12 | 0.184 | 0.567 | 28 | 0.209 | 0.286 |
| *A*11:05* |  |  |  | 9 | -0.083 | 0.831 |
| *A*11:06* |  |  |  | 9 | 0.325 | 0.394 |
| *A*11:10* |  |  |  | 5 | 0.544 | 0.343 |
| *A*11:12* |  |  |  | 7 | 0.650 | 0.114 |
| *A*23:01* | 24 | -0.084 | 0.696 | 57 | -0.059 | 0.661 |
| *A*23:02* |  |  |  | 8 | -0.101 | 0.813 |
| *A*23:03* |  |  |  | 9 | -0.289 | 0.451 |
| *A*23:04* | 5 | 0.622 | 0.262 | 11 | 0.013 | 0.969 |
| *A*23:05* | 5 | 0.705 | 0.184 | 11 | 0.409 | 0.212 |
| *A*24:02* | 24 | 0.072 | 0.740 | 55 | -0.063 | 0.646 |
| *A*24:03* | 19 | 0.281 | 0.243 | 38 | -0.019 | 0.912 |
| *A*24:04* | 9 | 0.334 | 0.379 | 26 | -0.039 | 0.850 |
| *A*24:05* | 9 | 0.395 | 0.292 | 24 | 0.147 | 0.492 |
| *A*24:06* | 14 | 0.123 | 0.676 | 30 | 0.139 | 0.464 |
| *A*24:07* | 13 | 0.165 | 0.591 | 29 | 0.909 | 0.000 |
| *A*24:08* | 9 | 0.396 | 0.291 | 23 | -0.035 | 0.874 |
| *A*24:10* | 12 | 0.417 | 0.178 | 26 | 0.176 | 0.391 |
| *A*24:13* | 13 | 0.334 | 0.264 | 30 | -0.181 | 0.339 |
| *A*24:14* | 9 | 0.361 | 0.339 | 22 | 0.200 | 0.373 |
| *A*24:15* |  |  |  | 10 | -0.254 | 0.480 |
| *A*24:17* |  |  |  | 12 | -0.209 | 0.515 |
| *A*24:18* |  |  |  | 11 | -0.329 | 0.324 |
| *A*24:20* |  |  |  | 9 | 0.538 | 0.135 |
| *A*24:21* |  |  |  | 7 | -0.076 | 0.871 |
| *A*24:22* |  |  |  | 8 | -0.084 | 0.843 |
| *A*24:23* |  |  |  | 10 | 0.223 | 0.537 |
| *A*24:24* |  |  |  | 9 | 0.497 | 0.173 |
| *A*24:26* |  |  |  | 7 | -0.411 | 0.360 |
| *A*24:27* |  |  |  | 7 | 0.167 | 0.721 |
| *A*24:28* |  |  |  | 6 | 0.800 | 0.056 |
| *A*24:29* |  |  |  | 5 | 0.802 | 0.103 |
| *A*24:31* |  |  |  | 7 | 0.785 | 0.036 |
| *A*25:01* | 17 | -0.290 | 0.259 | 47 | -0.261 | 0.076 |
| *A*25:02* | 9 | 0.562 | 0.115 | 23 | -0.173 | 0.431 |
| *A*25:04* |  |  |  | 5 | -0.375 | 0.534 |
| *A*26:01* | 26 | -0.288 | 0.153 | 61 | -0.346 | 0.006 |
| *A*26:02* | 9 | 0.463 | 0.209 | 22 | -0.427 | 0.048 |
| *A*26:03* | 9 | 0.373 | 0.323 | 24 | 0.037 | 0.865 |
| *A*26:04* |  |  |  | 22 | -0.182 | 0.419 |
| *A*26:05* | 9 | 0.319 | 0.403 | 24 | -0.045 | 0.835 |
| *A*26:06* |  |  |  | 21 | -0.176 | 0.445 |
| *A*26:07* | 8 | -0.252 | 0.548 | 20 | 0.069 | 0.773 |
| *A*26:08* | 15 | 0.040 | 0.888 | 32 | -0.062 | 0.735 |
| *A*26:09* | 9 | 0.319 | 0.403 | 22 | 0.073 | 0.748 |
| *A*26:10* |  |  |  | 10 | 0.348 | 0.324 |
| *A*26:12* | 9 | 0.469 | 0.203 | 17 | 0.421 | 0.092 |
| *A*26:14* |  |  |  | 8 | 0.469 | 0.242 |
| *A*26:15* |  |  |  | 6 | -0.400 | 0.432 |
| *A*26:16* |  |  |  | 5 | 0.802 | 0.103 |
| *A*26:18* |  |  |  | 5 | 0.523 | 0.365 |
| *A*29:01* | 18 | 0.134 | 0.597 | 48 | -0.050 | 0.738 |
| *A*29:02* | 20 | -0.326 | 0.160 | 46 | -0.092 | 0.541 |
| *A*29:03* | 10 | 0.532 | 0.113 | 21 | 0.087 | 0.707 |
| *A*29:04* |  |  |  | 7 | 0.167 | 0.721 |
| *A*29:10* |  |  |  | 5 | -0.598 | 0.287 |
| *A*30:01* | 23 | 0.316 | 0.142 | 56 | 0.109 | 0.425 |
| *A*30:02* | 20 | -0.142 | 0.551 | 50 | 0.076 | 0.601 |
| *A*30:03* | 13 | 0.317 | 0.291 | 26 | 0.250 | 0.218 |
| *A*30:04* | 16 | 0.017 | 0.949 | 37 | 0.308 | 0.064 |
| *A*30:06* | 7 | 0.300 | 0.514 | 12 | 0.280 | 0.378 |
| *A*30:08* | 5 | 0.686 | 0.201 | 11 | 0.583 | 0.060 |
| *A*30:09* |  |  |  | 9 | 0.778 | 0.013 |
| *A*30:10* |  |  |  | 6 | 0.207 | 0.694 |
| *A*30:11* |  |  |  | 5 | 0.875 | 0.052 |
| *A*30:12* |  |  |  | 5 | 0.875 | 0.052 |
| *A*31:01* | 5 | -0.840 | 0.075 | 11 | 0.022 | 0.949 |
| *A*31:02* |  |  |  | 11 | 0.109 | 0.751 |
| *A*31:03* | 8 | -0.216 | 0.607 | 16 | -0.115 | 0.671 |
| *A*31:04* | 7 | 0.358 | 0.431 | 13 | 0.559 | 0.047 |
| *A*31:05* |  |  |  | 7 | -0.612 | 0.144 |
| *A*31:06* |  |  |  | 5 | 0.875 | 0.052 |
| *A*31:08* |  |  |  | 9 | 0.618 | 0.076 |
| *A*31:09* |  |  |  | 6 | 0.640 | 0.171 |
| *A*32:01* | 26 | -0.379 | 0.056 | 61 | -0.269 | 0.036 |
| *A*32:02* | 10 | 0.368 | 0.295 | 23 | 0.050 | 0.822 |
| *A*32:04* |  |  |  | 8 | 0.349 | 0.397 |
| *A*32:05* |  |  |  | 5 | 0.802 | 0.103 |
| *A*32:06* |  |  |  | 7 | 0.116 | 0.805 |
| *A*33:01* | 19 | 0.173 | 0.478 | 50 | -0.194 | 0.178 |
| *A*33:03* | 21 | 0.337 | 0.135 | 50 | -0.005 | 0.972 |
| *A*33:04* |  |  |  | 8 | -0.084 | 0.843 |
| *A*33:05* |  |  |  | 10 | -0.373 | 0.288 |
| *A*34:01* | 15 | 0.430 | 0.109 | 33 | 0.710 | 0.000 |
| *A*34:02* | 19 | 0.112 | 0.649 | 42 | 0.292 | 0.061 |
| *A*34:03* |  |  |  | 6 | -0.297 | 0.567 |
| *A*34:05* |  |  |  | 6 | 0.536 | 0.273 |
| *A*36:01* | 20 | 0.323 | 0.165 | 41 | 0.152 | 0.344 |
| *A*36:03* |  |  |  | 5 | -0.404 | 0.500 |
| *A*43:01* |  |  |  | 31 | 0.132 | 0.479 |
| *A*66:01* | 20 | -0.196 | 0.408 | 53 | -0.045 | 0.748 |
| *A*66:02* | 17 | 0.198 | 0.446 | 31 | -0.057 | 0.761 |
| *A*66:03* | 12 | 0.687 | 0.014 | 24 | 0.061 | 0.776 |
| *A*68:01* | 20 | 0.162 | 0.496 | 42 | 0.080 | 0.614 |
| *A*68:02* | 24 | -0.125 | 0.560 | 54 | 0.332 | 0.014 |
| *A*68:03* | 9 | -0.193 | 0.619 | 23 | -0.135 | 0.540 |
| *A*68:04* | 9 | 0.556 | 0.120 | 21 | 0.453 | 0.039 |
| *A*68:05* | 8 | -0.252 | 0.548 | 20 | 0.054 | 0.822 |
| *A*68:06* |  |  |  | 12 | 0.155 | 0.631 |
| *A*68:07* |  |  |  | 9 | -0.083 | 0.831 |
| *A*68:08* |  |  |  | 10 | 0.147 | 0.686 |
| *A*68:12* |  |  |  | 11 | -0.276 | 0.411 |
| *A*68:13* |  |  |  | 9 | 0.481 | 0.190 |
| *A*68:15* | 6 | -0.318 | 0.540 | 11 | 0.260 | 0.440 |
| *A*68:24* |  |  |  | 5 | -0.612 | 0.272 |
| *A*69:01* | 20 | -0.238 | 0.312 | 42 | -0.170 | 0.282 |
| *A*74:01* | 20 | 0.310 | 0.183 | 34 | 0.378 | 0.028 |
| *A*74:02* |  |  |  | 8 | 0.061 | 0.887 |
| *A*74:03* | 9 | 0.036 | 0.927 | 20 | 0.229 | 0.330 |
| *A*80:01* | 22 | 0.326 | 0.139 | 40 | 0.268 | 0.094 |
| *B*07:02* | 20 | 0.126 | 0.597 | 49 | -0.150 | 0.304 |
| *B*07:03* |  |  |  | 20 | -0.188 | 0.427 |
| *B*07:04* |  |  |  | 23 | -0.358 | 0.093 |
| *B*07:05* | 21 | 0.260 | 0.256 | 49 | 0.251 | 0.082 |
| *B*07:06* | 8 | -0.323 | 0.434 | 22 | 0.133 | 0.556 |
| *B*07:07* | 9 | 0.844 | 0.004 | 22 | 0.567 | 0.006 |
| *B*07:08* | 8 | 0.912 | 0.002 | 20 | 0.313 | 0.179 |
| *B*07:09* |  |  |  | 20 | -0.313 | 0.179 |
| *B*07:10* |  |  |  | 20 | -0.205 | 0.387 |
| *B*07:12* | 8 | 0.315 | 0.447 | 18 | 0.347 | 0.158 |
| *B*07:13* | 7 | 0.395 | 0.381 | 16 | 0.389 | 0.136 |
| *B*07:14* |  |  |  | 6 | 0.397 | 0.436 |
| *B*07:15* |  |  |  | 6 | 0.652 | 0.161 |
| *B*07:17* |  |  |  | 6 | 0.603 | 0.205 |
| *B*07:20* |  |  |  | 14 | -0.367 | 0.197 |
| *B*07:22* |  |  |  | 5 | 0.802 | 0.103 |
| *B*07:26* |  |  |  | 7 | 0.167 | 0.721 |
| *B*08:01* | 25 | -0.451 | 0.024 | 59 | -0.134 | 0.313 |
| *B*08:02* |  |  |  | 21 | -0.162 | 0.484 |
| *B*08:03* | 9 | 0.842 | 0.004 | 25 | 0.174 | 0.406 |
| *B*08:04* | 9 | 0.813 | 0.008 | 22 | 0.230 | 0.304 |
| *B*08:05* | 9 | 0.581 | 0.101 | 22 | 0.313 | 0.156 |
| *B*08:09* |  |  |  | 6 | -0.400 | 0.432 |
| *B*08:12* |  |  |  | 6 | -0.087 | 0.870 |
| *B*08:18* |  |  |  | 5 | 0.015 | 0.981 |
| *B*13:01* | 14 | 0.336 | 0.240 | 39 | 0.009 | 0.955 |
| *B*13:02* | 21 | 0.260 | 0.256 | 51 | -0.148 | 0.301 |
| *B*13:03* | 13 | 0.174 | 0.570 | 28 | 0.190 | 0.333 |
| *B*13:04* | 10 | 0.638 | 0.047 | 22 | 0.596 | 0.003 |
| *B*13:09* |  |  |  | 5 | 0.875 | 0.052 |
| *B*13:11* |  |  |  | 5 | 0.875 | 0.052 |
| *B*14:01* | 17 | 0.032 | 0.903 | 45 | 0.014 | 0.926 |
| *B*14:02* | 21 | -0.465 | 0.034 | 52 | -0.056 | 0.693 |
| *B*14:03* | 13 | 0.126 | 0.683 | 25 | 0.205 | 0.325 |
| *B*14:04* | 8 | 0.315 | 0.447 | 20 | -0.119 | 0.618 |
| *B*14:05* | 9 | 0.820 | 0.007 | 21 | 0.462 | 0.035 |
| *B*14:06* |  |  |  | 5 | -0.404 | 0.500 |
| *B*15:01* | 23 | -0.082 | 0.710 | 50 | -0.332 | 0.018 |
| *B*15:02* | 12 | 0.354 | 0.258 | 31 | 0.511 | 0.003 |
| *B*15:03* | 21 | -0.091 | 0.696 | 48 | 0.122 | 0.408 |
| *B*15:04* | 11 | 0.302 | 0.367 | 22 | 0.016 | 0.942 |
| *B*15:05* | 9 | 0.352 | 0.353 | 22 | 0.590 | 0.004 |
| *B*15:06* | 8 | 0.763 | 0.028 | 22 | 0.233 | 0.297 |
| *B*15:07* | 9 | 0.062 | 0.874 | 26 | 0.093 | 0.653 |
| *B*15:08* | 10 | -0.038 | 0.917 | 28 | 0.290 | 0.134 |
| *B*15:09* | 14 | 0.321 | 0.263 | 30 | 0.143 | 0.451 |
| *B*15:10* | 21 | 0.144 | 0.534 | 38 | 0.301 | 0.067 |
| *B*15:11* | 10 | 0.169 | 0.640 | 24 | 0.104 | 0.629 |
| *B*15:12* | 10 | 0.029 | 0.938 | 22 | 0.181 | 0.421 |
| *B*15:13* | 10 | 0.262 | 0.464 | 23 | 0.447 | 0.032 |
| *B*15:14* |  |  |  | 21 | -0.178 | 0.439 |
| *B*15:15* | 11 | -0.307 | 0.358 | 23 | -0.068 | 0.759 |
| *B*15:16* | 18 | 0.101 | 0.691 | 35 | 0.366 | 0.031 |
| *B*15:17* | 21 | -0.469 | 0.032 | 47 | 0.106 | 0.479 |
| *B*15:18* | 21 | 0.297 | 0.191 | 43 | 0.074 | 0.638 |
| *B*15:20* | 8 | -0.006 | 0.988 | 17 | 0.237 | 0.360 |
| *B*15:21* | 11 | 0.529 | 0.094 | 26 | 0.170 | 0.405 |
| *B*15:23* | 8 | 0.710 | 0.048 | 20 | 0.384 | 0.095 |
| *B*15:24* | 7 | -0.093 | 0.843 | 21 | 0.137 | 0.553 |
| *B*15:25* | 12 | 0.814 | 0.001 | 27 | 0.406 | 0.035 |
| *B*15:27* | 9 | 0.551 | 0.124 | 21 | -0.191 | 0.406 |
| *B*15:28* | 8 | 0.861 | 0.006 | 20 | 0.216 | 0.361 |
| *B*15:29* | 8 | 0.730 | 0.040 | 21 | 0.388 | 0.082 |
| *B*15:30* | 7 | 0.161 | 0.730 | 18 | 0.273 | 0.272 |
| *B*15:31* | 13 | 0.413 | 0.161 | 25 | -0.220 | 0.291 |
| *B*15:32* | 9 | 0.512 | 0.159 | 21 | 0.268 | 0.241 |
| *B*15:33* |  |  |  | 18 | -0.258 | 0.302 |
| *B*15:34* | 7 | 0.395 | 0.381 | 20 | 0.054 | 0.821 |
| *B*15:35* | 7 | 0.880 | 0.009 | 19 | 0.513 | 0.025 |
| *B*15:36* | 9 | 0.760 | 0.017 | 22 | 0.423 | 0.050 |
| *B*15:37* | 14 | -0.007 | 0.982 | 25 | 0.369 | 0.070 |
| *B*15:38* | 7 | 0.116 | 0.804 | 18 | 0.407 | 0.094 |
| *B*15:39* | 6 | 0.211 | 0.689 | 19 | 0.032 | 0.898 |
| *B*15:40* | 8 | 0.639 | 0.088 | 19 | 0.446 | 0.056 |
| *B*15:42* |  |  |  | 8 | -0.290 | 0.486 |
| *B*15:45* | 7 | 0.395 | 0.381 | 18 | 0.396 | 0.104 |
| *B*15:46* | 7 | 0.395 | 0.381 | 19 | -0.030 | 0.903 |
| *B*15:47* | 7 | 0.395 | 0.381 | 18 | 0.241 | 0.335 |
| *B*15:48* |  |  |  | 7 | 0.801 | 0.031 |
| *B*15:50* |  |  |  | 6 | 0.800 | 0.056 |
| *B*15:52* |  |  |  | 6 | -0.297 | 0.567 |
| *B*15:53* |  |  |  | 6 | 0.800 | 0.056 |
| *B*15:54* |  |  |  | 6 | -0.297 | 0.567 |
| *B*15:55* | 7 | 0.256 | 0.580 | 12 | 0.386 | 0.215 |
| *B*15:56* |  |  |  | 6 | 0.800 | 0.056 |
| *B*15:58* |  |  |  | 7 | 0.660 | 0.107 |
| *B*15:61* |  |  |  | 5 | -0.319 | 0.601 |
| *B*15:67* |  |  |  | 6 | 0.635 | 0.175 |
| *B*15:68* |  |  |  | 5 | -0.694 | 0.194 |
| *B*15:73* |  |  |  | 5 | -0.375 | 0.534 |
| *B*18:01* | 23 | -0.428 | 0.041 | 57 | -0.046 | 0.733 |
| *B*18:02* | 11 | 0.632 | 0.037 | 25 | 0.381 | 0.061 |
| *B*18:03* | 11 | 0.663 | 0.026 | 29 | -0.027 | 0.889 |
| *B*18:04* | 8 | 0.912 | 0.002 |  |  |  |
| *B*18:05* |  |  |  | 19 | -0.185 | 0.449 |
| *B*18:07* | 10 | 0.601 | 0.066 | 22 | 0.349 | 0.111 |
| *B*18:08* |  |  |  | 7 | 0.167 | 0.721 |
| *B*18:11* |  |  |  | 6 | 0.108 | 0.838 |
| *B*18:14* |  |  |  | 6 | 0.113 | 0.832 |
| *B*18:18* |  |  |  | 6 | -0.293 | 0.573 |
| *B*27:01* | 7 | 0.395 | 0.381 | 19 | -0.023 | 0.926 |
| *B*27:02* | 10 | -0.364 | 0.301 | 33 | -0.070 | 0.699 |
| *B*27:03* | 17 | 0.598 | 0.011 | 32 | 0.364 | 0.041 |
| *B*27:04* | 10 | 0.903 | 0.000 | 30 | 0.149 | 0.432 |
| *B*27:05* | 18 | -0.038 | 0.880 | 48 | -0.352 | 0.014 |
| *B*27:06* | 10 | -0.080 | 0.826 | 24 | 0.797 | 0.000 |
| *B*27:07* | 9 | 0.655 | 0.055 | 26 | -0.020 | 0.921 |
| *B*27:08* | 8 | 0.093 | 0.826 | 24 | 0.103 | 0.631 |
| *B*27:09* |  |  |  | 21 | -0.285 | 0.210 |
| *B*27:11* | 7 | 0.395 | 0.381 | 20 | 0.267 | 0.255 |
| *B*27:12* | 7 | 0.395 | 0.381 | 21 | 0.286 | 0.208 |
| *B*27:13* |  |  |  | 8 | 0.231 | 0.583 |
| *B*27:14* |  |  |  | 21 | -0.228 | 0.321 |
| *B*27:20* |  |  |  | 6 | -0.297 | 0.567 |
| *B*27:21* |  |  |  | 5 | 0.802 | 0.103 |
| *B*35:01* | 22 | -0.020 | 0.928 | 53 | -0.096 | 0.496 |
| *B*35:02* | 15 | -0.242 | 0.385 | 40 | -0.368 | 0.020 |
| *B*35:03* | 17 | -0.357 | 0.160 | 47 | -0.228 | 0.124 |
| *B*35:04* | 8 | 0.185 | 0.662 | 22 | 0.083 | 0.714 |
| *B*35:05* | 14 | 0.254 | 0.381 | 31 | 0.428 | 0.016 |
| *B*35:06* | 9 | 0.324 | 0.395 | 22 | 0.196 | 0.382 |
| *B*35:08* | 15 | -0.464 | 0.082 | 36 | -0.011 | 0.950 |
| *B*35:09* | 7 | 0.367 | 0.418 | 16 | 0.163 | 0.545 |
| *B*35:10* | 8 | 0.861 | 0.006 | 21 | 0.219 | 0.340 |
| *B*35:11* | 6 | 0.616 | 0.192 | 17 | 0.061 | 0.815 |
| *B*35:12* | 9 | 0.012 | 0.976 | 22 | 0.218 | 0.330 |
| *B*35:13* | 8 | 0.755 | 0.030 | 21 | 0.077 | 0.741 |
| *B*35:14* | 7 | -0.214 | 0.645 | 20 | 0.081 | 0.733 |
| *B*35:15* | 9 | 0.535 | 0.138 | 21 | 0.539 | 0.012 |
| *B*35:16* | 8 | 0.061 | 0.886 | 20 | 0.231 | 0.327 |
| *B*35:17* | 8 | 0.319 | 0.442 | 20 | 0.288 | 0.218 |
| *B*35:18* | 8 | -0.112 | 0.793 | 20 | 0.101 | 0.671 |
| *B*35:19* | 8 | -0.112 | 0.793 | 21 | 0.030 | 0.899 |
| *B*35:20* | 11 | 0.590 | 0.056 | 25 | -0.124 | 0.556 |
| *B*35:21* | 9 | 0.678 | 0.045 | 22 | 0.060 | 0.791 |
| *B*35:23* | 6 | -0.137 | 0.795 | 17 | 0.115 | 0.661 |
| *B*35:24* | 6 | -0.137 | 0.795 | 17 | 0.133 | 0.610 |
| *B*35:25* | 8 | 0.861 | 0.006 | 20 | 0.354 | 0.126 |
| *B*35:27* |  |  |  | 11 | -0.365 | 0.270 |
| *B*35:28* | 7 | 0.536 | 0.215 | 16 | 0.417 | 0.109 |
| *B*35:30* | 7 | 0.480 | 0.275 | 16 | 0.303 | 0.255 |
| *B*35:32* | 6 | 0.616 | 0.192 | 15 | 0.452 | 0.091 |
| *B*35:33* |  |  |  | 16 | -0.207 | 0.442 |
| *B*35:34* |  |  |  | 8 | 0.085 | 0.842 |
| *B*35:43* |  |  |  | 5 | 0.441 | 0.458 |
| *B*37:01* | 19 | 0.129 | 0.600 | 47 | -0.150 | 0.314 |
| *B*37:02* |  |  |  | 22 | -0.183 | 0.415 |
| *B*37:05* |  |  |  | 6 | 0.108 | 0.838 |
| *B*38:01* | 19 | -0.150 | 0.541 | 48 | -0.200 | 0.174 |
| *B*38:02* | 9 | 0.874 | 0.002 | 27 | 0.313 | 0.112 |
| *B*38:04* |  |  |  | 8 | 0.771 | 0.025 |
| *B*38:05* |  |  |  | 6 | 0.800 | 0.056 |
| *B*38:06* |  |  |  | 7 | -0.084 | 0.859 |
| *B*38:09* |  |  |  | 7 | -0.331 | 0.468 |
| *B*39:01* | 17 | 0.166 | 0.525 | 49 | -0.177 | 0.225 |
| *B*39:02* | 9 | -0.106 | 0.786 | 21 | 0.069 | 0.767 |
| *B*39:03* | 14 | -0.023 | 0.939 | 27 | 0.159 | 0.428 |
| *B*39:04* | 9 | 0.123 | 0.752 | 23 | 0.167 | 0.446 |
| *B*39:05* | 10 | 0.009 | 0.981 | 23 | 0.396 | 0.061 |
| *B*39:06* | 11 | -0.093 | 0.785 | 31 | 0.135 | 0.469 |
| *B*39:07* | 7 | 0.395 | 0.381 | 18 | 0.171 | 0.497 |
| *B*39:08* | 7 | -0.212 | 0.648 | 19 | 0.270 | 0.264 |
| *B*39:09* | 11 | 0.195 | 0.566 | 24 | 0.414 | 0.044 |
| *B*39:10* | 17 | 0.247 | 0.339 | 34 | 0.352 | 0.041 |
| *B*39:11* | 7 | 0.395 | 0.381 | 19 | 0.241 | 0.321 |
| *B*39:12* | 7 | 0.116 | 0.804 | 19 | 0.202 | 0.407 |
| *B*39:13* |  |  |  | 7 | -0.294 | 0.522 |
| *B*39:14* | 8 | 0.291 | 0.484 | 18 | 0.319 | 0.197 |
| *B*39:15* | 10 | 0.675 | 0.032 | 20 | 0.264 | 0.260 |
| *B*39:23* |  |  |  | 6 | -0.479 | 0.337 |
| *B*39:24* | 8 | 0.364 | 0.375 | 20 | -0.011 | 0.962 |
| *B*40:01* | 21 | 0.313 | 0.167 | 58 | -0.429 | 0.001 |
| *B*40:02* | 21 | 0.021 | 0.927 | 54 | 0.121 | 0.382 |
| *B*40:03* | 8 | 0.210 | 0.618 | 23 | 0.312 | 0.147 |
| *B*40:04* | 9 | 0.250 | 0.517 | 23 | 0.290 | 0.179 |
| *B*40:05* | 8 | 0.072 | 0.866 | 23 | 0.122 | 0.578 |
| *B*40:06* | 12 | -0.194 | 0.547 | 34 | 0.077 | 0.665 |
| *B*40:07* | 7 | 0.395 | 0.381 | 22 | -0.160 | 0.478 |
| *B*40:08* | 8 | 0.734 | 0.038 | 23 | 0.103 | 0.639 |
| *B*40:09* | 8 | 0.233 | 0.578 | 21 | 0.237 | 0.302 |
| *B*40:10* | 9 | 0.773 | 0.015 | 27 | -0.099 | 0.622 |
| *B*40:11* | 8 | 0.628 | 0.096 | 23 | -0.070 | 0.752 |
| *B*40:12* | 13 | 0.463 | 0.111 | 28 | 0.075 | 0.704 |
| *B*40:14* | 7 | 0.263 | 0.569 | 21 | 0.208 | 0.366 |
| *B*40:15* |  |  |  | 21 | -0.176 | 0.445 |
| *B*40:16* | 14 | 0.233 | 0.423 | 27 | 0.373 | 0.055 |
| *B*40:18* | 7 | 0.395 | 0.381 | 20 | 0.391 | 0.088 |
| *B*40:19* |  |  |  | 21 | -0.064 | 0.782 |
| *B*40:20* | 7 | 0.395 | 0.381 | 19 | 0.298 | 0.215 |
| *B*40:21* |  |  |  | 6 | 0.800 | 0.056 |
| *B*40:23* |  |  |  | 5 | 0.749 | 0.145 |
| *B*40:27* |  |  |  | 6 | 0.321 | 0.535 |
| *B*40:35* |  |  |  | 5 | 0.802 | 0.103 |
| *B*40:40* |  |  |  | 5 | -0.736 | 0.156 |
| *B*40:42* |  |  |  | 5 | 0.751 | 0.143 |
| *B*41:01* | 20 | 0.062 | 0.794 | 47 | 0.083 | 0.580 |
| *B*41:02* | 17 | 0.250 | 0.333 | 43 | -0.033 | 0.836 |
| *B*41:03* | 10 | 0.806 | 0.005 | 22 | 0.368 | 0.092 |
| *B*42:01* | 22 | 0.057 | 0.802 | 44 | 0.253 | 0.097 |
| *B*42:02* | 17 | 0.335 | 0.188 | 34 | 0.376 | 0.028 |
| *B*44:02* | 19 | -0.029 | 0.905 | 54 | -0.288 | 0.035 |
| *B*44:03* | 20 | -0.023 | 0.922 | 49 | -0.217 | 0.135 |
| *B*44:04* | 8 | 0.871 | 0.005 | 26 | 0.234 | 0.250 |
| *B*44:05* | 13 | 0.128 | 0.677 | 35 | -0.243 | 0.159 |
| *B*44:06* | 8 | 0.841 | 0.009 | 23 | 0.344 | 0.108 |
| *B*44:07* | 14 | 0.096 | 0.744 | 28 | 0.227 | 0.246 |
| *B*44:08* | 8 | 0.861 | 0.006 | 20 | 0.147 | 0.537 |
| *B*44:09* | 8 | 0.664 | 0.073 | 20 | 0.404 | 0.078 |
| *B*44:10* | 9 | 0.743 | 0.022 | 21 | 0.057 | 0.806 |
| *B*44:12* |  |  |  | 7 | 0.801 | 0.031 |
| *B*44:15* | 10 | 0.217 | 0.547 | 18 | 0.336 | 0.173 |
| *B*44:18* |  |  |  | 7 | -0.063 | 0.893 |
| *B*44:21* |  |  |  | 6 | -0.400 | 0.432 |
| *B*44:22* |  |  |  | 5 | 0.802 | 0.103 |
| *B*44:26* |  |  |  | 5 | 0.559 | 0.327 |
| *B*44:27* |  |  |  | 5 | -0.488 | 0.405 |
| *B*44:29* |  |  |  | 5 | -0.375 | 0.534 |
| *B*45:01* | 22 | 0.183 | 0.415 | 52 | 0.265 | 0.058 |
| *B*45:02* | 8 | 0.751 | 0.032 | 20 | 0.119 | 0.618 |
| *B*45:04* |  |  |  | 6 | 0.603 | 0.205 |
| *B*46:01* | 9 | 0.629 | 0.069 | 25 | -0.375 | 0.064 |
| *B*46:02* |  |  |  | 5 | 0.802 | 0.103 |
| *B*47:01* | 18 | -0.005 | 0.983 | 43 | -0.035 | 0.823 |
| *B*47:02* | 10 | 0.564 | 0.090 |  |  |  |
| *B*47:03* | 13 | 0.539 | 0.058 | 24 | 0.080 | 0.711 |
| *B*48:01* | 16 | 0.112 | 0.679 | 41 | 0.040 | 0.806 |
| *B*48:02* | 12 | 0.666 | 0.018 | 22 | 0.584 | 0.004 |
| *B*48:03* | 9 | 0.012 | 0.976 | 20 | -0.334 | 0.151 |
| *B*48:04* | 8 | 0.861 | 0.006 | 19 | 0.104 | 0.670 |
| *B*48:05* |  |  |  | 6 | -0.400 | 0.432 |
| *B*48:06* |  |  |  | 5 | 0.802 | 0.103 |
| *B*48:07* |  |  |  | 5 | 0.091 | 0.884 |
| *B*49:01* | 21 | 0.212 | 0.357 | 52 | -0.149 | 0.291 |
| *B*49:02* |  |  |  | 6 | 0.800 | 0.056 |
| *B*49:03* |  |  |  | 7 | 0.581 | 0.171 |
| *B*50:01* | 20 | -0.333 | 0.152 | 52 | 0.083 | 0.561 |
| *B*50:02* | 9 | -0.384 | 0.308 | 28 | 0.057 | 0.772 |
| *B*50:04* |  |  |  | 6 | -0.400 | 0.432 |
| *B*51:01* | 20 | -0.204 | 0.389 | 50 | -0.231 | 0.107 |
| *B*51:02* | 8 | 0.838 | 0.009 | 20 | 0.550 | 0.012 |
| *B*51:03* | 9 | 0.929 | 0.000 | 22 | 0.376 | 0.085 |
| *B*51:04* | 9 | 0.606 | 0.084 | 25 | 0.275 | 0.184 |
| *B*51:05* |  |  |  | 23 | 0.062 | 0.778 |
| *B*51:06* | 11 | 0.597 | 0.052 | 25 | 0.526 | 0.007 |
| *B*51:07* | 9 | 0.515 | 0.156 | 26 | 0.197 | 0.334 |
| *B*51:08* | 14 | 0.200 | 0.492 | 36 | -0.079 | 0.645 |
| *B*51:09* | 6 | 0.616 | 0.192 | 21 | -0.023 | 0.921 |
| *B*51:10* | 8 | 0.678 | 0.065 | 21 | 0.457 | 0.037 |
| *B*51:12* |  |  |  | 8 | 0.582 | 0.130 |
| *B*51:14* |  |  |  | 20 | -0.193 | 0.416 |
| *B*51:15* | 8 | 0.861 | 0.006 | 22 | -0.150 | 0.505 |
| *B*51:18* |  |  |  | 7 | 0.167 | 0.721 |
| *B*51:21* |  |  |  | 6 | 0.800 | 0.056 |
| *B*51:22* |  |  |  | 7 | 0.721 | 0.068 |
| *B*51:26* |  |  |  | 5 | -0.375 | 0.534 |
| *B*51:32* |  |  |  | 5 | -0.375 | 0.534 |
| *B*51:33* |  |  |  | 5 | 0.559 | 0.327 |
| *B*52:01* | 18 | -0.110 | 0.664 | 46 | -0.024 | 0.875 |
| *B*52:02* |  |  |  | 7 | -0.292 | 0.525 |
| *B*53:01* | 24 | 0.340 | 0.104 | 54 | 0.012 | 0.931 |
| *B*53:02* | 10 | 0.909 | 0.000 | 24 | 0.368 | 0.077 |
| *B*53:03* | 9 | 0.910 | 0.001 | 21 | 0.427 | 0.053 |
| *B*53:04* | 7 | 0.945 | 0.001 | 18 | 0.345 | 0.161 |
| *B*53:05* |  |  |  | 10 | 0.486 | 0.154 |
| *B*53:07* |  |  |  | 7 | 0.581 | 0.171 |
| *B*53:08* |  |  |  | 6 | 0.779 | 0.068 |
| *B*54:01* | 11 | 0.627 | 0.039 | 31 | -0.455 | 0.010 |
| *B*54:02* |  |  |  | 5 | 0.802 | 0.103 |
| *B*55:01* | 14 | -0.165 | 0.573 | 42 | -0.230 | 0.142 |
| *B*55:02* | 10 | 0.126 | 0.728 | 23 | -0.339 | 0.114 |
| *B*55:03* |  |  |  | 17 | -0.075 | 0.774 |
| *B*55:04* | 8 | 0.626 | 0.097 | 19 | -0.487 | 0.035 |
| *B*55:07* |  |  |  | 7 | 0.310 | 0.499 |
| *B*55:08* |  |  |  | 5 | 0.802 | 0.103 |
| *B*55:10* |  |  |  | 6 | 0.800 | 0.056 |
| *B*55:12* |  |  |  | 5 | 0.585 | 0.300 |
| *B*56:01* | 20 | 0.437 | 0.054 | 48 | -0.023 | 0.876 |
| *B*56:02* | 9 | 0.920 | 0.000 | 23 | -0.281 | 0.194 |
| *B*56:03* | 7 | 0.939 | 0.002 | 18 | 0.302 | 0.223 |
| *B*56:04* | 8 | 0.738 | 0.037 | 19 | -0.270 | 0.263 |
| *B*56:05* | 7 | 0.908 | 0.005 | 16 | 0.389 | 0.136 |
| *B*56:06* |  |  |  | 6 | 0.800 | 0.056 |
| *B*56:07* |  |  |  | 7 | 0.843 | 0.017 |
| *B*56:09* |  |  |  | 6 | 0.878 | 0.021 |
| *B*56:11* |  |  |  | 5 | 0.875 | 0.052 |
| *B*57:01* | 16 | 0.217 | 0.420 | 47 | -0.183 | 0.218 |
| *B*57:02* | 15 | 0.770 | 0.001 | 27 | 0.722 | 0.000 |
| *B*57:03* | 17 | 0.525 | 0.030 | 33 | 0.252 | 0.157 |
| *B*57:04* | 7 | 0.935 | 0.002 | 16 | 0.248 | 0.354 |
| *B*57:05* |  |  |  | 16 | -0.229 | 0.394 |
| *B*57:06* |  |  |  | 7 | -0.320 | 0.484 |
| *B*58:01* | 25 | -0.016 | 0.941 | 60 | 0.264 | 0.041 |
| *B*58:02* | 17 | -0.060 | 0.819 | 32 | 0.282 | 0.117 |
| *B*58:06* |  |  |  | 6 | 0.800 | 0.056 |
| *B*59:01* | 6 | 0.616 | 0.192 | 21 | -0.366 | 0.103 |
| *B*67:01* | 12 | -0.096 | 0.766 | 28 | 0.053 | 0.788 |
| *B*67:02* |  |  |  | 5 | -0.319 | 0.601 |
| *B*73:01* | 15 | 0.006 | 0.984 | 37 | -0.075 | 0.658 |
| *B*78:01* | 18 | 0.497 | 0.036 | 31 | 0.382 | 0.034 |
| *B*78:02* | 8 | 0.849 | 0.008 | 16 | 0.502 | 0.047 |
| *B*78:03* | 7 | 0.908 | 0.005 | 16 | 0.056 | 0.838 |
| *B*78:05* |  |  |  | 6 | 0.108 | 0.838 |
| *B*81:01* | 16 | -0.102 | 0.706 | 29 | 0.530 | 0.003 |
| *B*81:02* |  |  |  | 5 | -0.630 | 0.255 |
| *B*82:01* | 13 | 0.306 | 0.310 | 22 | 0.590 | 0.004 |
| *B*82:02* | 5 | 0.848 | 0.069 | 9 | 0.595 | 0.091 |
| *C*01:02* | 17 | -0.006 | 0.981 | 46 | -0.325 | 0.027 |
| *C*01:03* | 6 | -0.089 | 0.868 | 18 | -0.572 | 0.013 |
| *C*01:04* |  |  |  | 10 | -0.101 | 0.781 |
| *C*02:02* | 12 | 0.310 | 0.327 | 36 | -0.215 | 0.209 |
| *C*02:03* |  |  |  | 12 | -0.185 | 0.565 |
| *C*03:02* | 19 | 0.008 | 0.975 | 45 | 0.160 | 0.295 |
| *C*03:03* | 21 | 0.037 | 0.872 | 51 | -0.077 | 0.592 |
| *C*03:04* | 19 | 0.040 | 0.871 | 42 | -0.003 | 0.984 |
| *C*03:05* | 8 | -0.312 | 0.451 | 16 | 0.281 | 0.291 |
| *C*03:06* |  |  |  | 11 | 0.201 | 0.553 |
| *C*03:07* |  |  |  | 11 | -0.529 | 0.094 |
| *C*03:08* |  |  |  | 13 | 0.223 | 0.463 |
| *C*03:09* |  |  |  | 9 | 0.707 | 0.033 |
| *C*03:10* |  |  |  | 10 | 0.430 | 0.214 |
| *C*03:12* |  |  |  | 9 | 0.433 | 0.244 |
| *C*04:01* | 19 | 0.153 | 0.532 | 49 | -0.017 | 0.909 |
| *C*04:03* | 9 | 0.094 | 0.810 | 27 | 0.170 | 0.398 |
| *C*04:04* | 10 | 0.294 | 0.410 | 21 | -0.038 | 0.872 |
| *C*04:05* |  |  |  | 11 | 0.115 | 0.736 |
| *C*04:06* |  |  |  | 13 | 0.173 | 0.571 |
| *C*04:07* | 11 | -0.061 | 0.858 | 24 | 0.317 | 0.132 |
| *C*05:01* | 13 | -0.616 | 0.025 | 39 | -0.310 | 0.055 |
| *C*05:03* |  |  |  | 7 | 0.070 | 0.881 |
| *C*05:04* |  |  |  | 9 | -0.350 | 0.356 |
| *C*06:02* | 19 | -0.615 | 0.005 | 48 | 0.105 | 0.477 |
| *C*06:03* |  |  |  | 12 | 0.520 | 0.083 |
| *C*06:04* |  |  |  | 15 | -0.009 | 0.973 |
| *C*06:06* |  |  |  | 6 | 0.698 | 0.123 |
| *C*07:01* | 20 | 0.134 | 0.572 | 45 | -0.035 | 0.821 |
| *C*07:02* | 20 | 0.115 | 0.629 | 47 | 0.081 | 0.590 |
| *C*07:03* | 6 | 0.056 | 0.916 | 17 | -0.136 | 0.602 |
| *C*07:04* | 18 | -0.293 | 0.239 | 41 | 0.091 | 0.572 |
| *C*07:05* | 11 | 0.131 | 0.700 | 21 | 0.158 | 0.495 |
| *C*07:06* |  |  |  | 13 | 0.288 | 0.340 |
| *C*07:07* | 5 | 0.203 | 0.743 | 17 | 0.171 | 0.512 |
| *C*07:08* | 6 | 0.199 | 0.705 | 14 | 0.689 | 0.006 |
| *C*07:12* |  |  |  | 8 | -0.339 | 0.411 |
| *C*07:13* |  |  |  | 10 | -0.081 | 0.824 |
| *C*07:14* | 6 | 0.368 | 0.473 | 12 | 0.694 | 0.012 |
| *C*07:15* |  |  |  | 5 | -0.559 | 0.327 |
| *C*07:17* |  |  |  | 5 | 0.829 | 0.083 |
| *C*07:19* |  |  |  | 5 | 0.015 | 0.981 |
| *C*07:21* |  |  |  | 5 | 0.015 | 0.981 |
| *C*07:22* |  |  |  | 5 | -0.598 | 0.287 |
| *C*08:01* | 17 | -0.293 | 0.255 | 37 | 0.068 | 0.689 |
| *C*08:02* | 15 | -0.145 | 0.605 | 41 | -0.156 | 0.332 |
| *C*08:03* | 5 | -0.028 | 0.965 | 17 | 0.158 | 0.544 |
| *C*08:04* | 9 | -0.162 | 0.677 | 17 | 0.264 | 0.305 |
| *C*08:05* |  |  |  | 11 | -0.389 | 0.237 |
| *C*08:06* |  |  |  | 11 | 0.181 | 0.595 |
| *C*08:07* |  |  |  | 6 | -0.542 | 0.266 |
| *C*12:02* | 16 | -0.276 | 0.300 | 45 | 0.062 | 0.684 |
| *C*12:03* | 19 | 0.232 | 0.340 | 50 | -0.222 | 0.122 |
| *C*12:05* |  |  |  | 12 | -0.366 | 0.242 |
| *C*12:07* |  |  |  | 8 | -0.424 | 0.296 |
| *C*14:02* | 16 | -0.222 | 0.409 | 40 | -0.112 | 0.490 |
| *C*14:03* | 14 | -0.107 | 0.716 | 29 | -0.123 | 0.524 |
| *C*14:04* |  |  |  | 11 | 0.435 | 0.181 |
| *C*14:05* |  |  |  | 5 | -0.612 | 0.272 |
| *C*15:02* | 16 | -0.365 | 0.164 | 42 | -0.157 | 0.321 |
| *C*15:03* | 10 | 0.385 | 0.272 | 20 | 0.385 | 0.093 |
| *C*15:04* | 8 | -0.099 | 0.815 | 22 | 0.290 | 0.191 |
| *C*15:05* | 15 | -0.197 | 0.481 | 34 | 0.053 | 0.768 |
| *C*15:06* |  |  |  | 11 | -0.389 | 0.237 |
| *C*15:07* | 7 | 0.673 | 0.097 | 17 | -0.140 | 0.592 |
| *C*15:08* |  |  |  | 13 | 0.232 | 0.446 |
| *C*15:09* |  |  |  | 6 | -0.612 | 0.197 |
| *C*16:01* |  |  |  | 43 | -0.023 | 0.882 |
| *C*16:02* | 14 | -0.416 | 0.139 | 39 | -0.056 | 0.735 |
| *C*16:04* | 9 | 0.324 | 0.395 | 19 | -0.195 | 0.423 |
| *C*17:01* | 14 | 0.178 | 0.543 | 36 | 0.159 | 0.355 |
| *C*17:02* |  |  |  | 7 | 0.023 | 0.961 |
| *C*17:03* |  |  |  | 8 | 0.268 | 0.521 |
| *C*18:01* | 12 | 0.422 | 0.172 | 24 | 0.266 | 0.208 |
| *C*18:02* |  |  |  | 6 | 0.000 | 1.000 |
| *DPA1*01:03* | 12 | -0.526 | 0.079 | 22 | -0.295 | 0.182 |
| *DPA1*01:04* |  |  |  | 16 | 0.156 | 0.564 |
| *DPA1*01:05* |  |  |  | 6 | -0.352 | 0.494 |
| *DPA1*02:01* |  |  |  | 7 | 0.099 | 0.832 |
| *DPA1*02:02* |  |  |  | 8 | -0.402 | 0.323 |
| *DPA1*02:03* |  |  |  | 6 | 0.192 | 0.715 |
| *DPA1*03:01* | 11 | 0.272 | 0.418 | 22 | 0.334 | 0.129 |
| *DPA1*03:02* | 7 | 0.076 | 0.871 | 11 | 0.322 | 0.334 |
| *DPA1*04:01* | 10 | 0.316 | 0.374 | 23 | 0.584 | 0.003 |
| *DPB1*01:01* | 16 | 0.650 | 0.006 | 44 | 0.175 | 0.255 |
| *DPB1*02:01* | 19 | 0.488 | 0.034 | 46 | -0.151 | 0.317 |
| *DPB1*02:02* | 13 | -0.282 | 0.351 | 39 | 0.152 | 0.355 |
| *DPB1*03:01* | 22 | -0.163 | 0.468 | 53 | -0.186 | 0.183 |
| *DPB1*04:01* | 23 | -0.116 | 0.599 | 56 | 0.003 | 0.982 |
| *DPB1*04:02* | 23 | -0.264 | 0.223 | 55 | 0.004 | 0.978 |
| *DPB1*05:01* | 16 | -0.229 | 0.393 | 47 | -0.187 | 0.208 |
| *DPB1*06:01* | 14 | -0.207 | 0.477 | 36 | -0.429 | 0.009 |
| *DPB1*08:01* | 10 | -0.196 | 0.587 | 26 | -0.004 | 0.983 |
| *DPB1*09:01* | 15 | -0.674 | 0.006 | 40 | -0.198 | 0.221 |
| *DPB1*10:01* | 16 | -0.624 | 0.010 | 41 | -0.115 | 0.474 |
| *DPB1*11:01* | 14 | -0.250 | 0.389 | 35 | -0.034 | 0.846 |
| *DPB1*13:01* | 21 | -0.321 | 0.155 | 52 | 0.389 | 0.004 |
| *DPB1*14:01* | 20 | 0.134 | 0.573 | 50 | 0.178 | 0.216 |
| *DPB1*15:01* | 17 | -0.414 | 0.099 | 43 | 0.039 | 0.805 |
| *DPB1*16:01* | 17 | -0.161 | 0.536 | 39 | -0.212 | 0.195 |
| *DPB1*17:01* | 21 | 0.346 | 0.124 | 45 | 0.046 | 0.764 |
| *DPB1*18:01* | 16 | -0.390 | 0.135 | 30 | 0.001 | 0.994 |
| *DPB1*19:01* | 16 | -0.067 | 0.806 | 43 | -0.057 | 0.714 |
| *DPB1*20:01* | 14 | -0.319 | 0.266 | 29 | -0.417 | 0.025 |
| *DPB1*21:01* | 11 | -0.595 | 0.053 | 24 | 0.100 | 0.643 |
| *DPB1*22:01* | 11 | -0.478 | 0.137 | 20 | -0.304 | 0.193 |
| *DPB1*23:01* | 17 | -0.775 | 0.000 | 35 | -0.114 | 0.513 |
| *DPB1*24:01* | 10 | -0.486 | 0.154 | 22 | -0.063 | 0.779 |
| *DPB1*25:01* | 12 | -0.698 | 0.012 | 22 | -0.209 | 0.349 |
| *DPB1*26:01* | 11 | 0.027 | 0.938 | 23 | 0.124 | 0.572 |
| *DPB1*27:01* | 16 | -0.410 | 0.115 | 27 | 0.256 | 0.198 |
| *DPB1*28:01* | 11 | -0.752 | 0.008 | 25 | 0.220 | 0.290 |
| *DPB1*29:01* | 12 | 0.193 | 0.548 | 27 | 0.256 | 0.198 |
| *DPB1*30:01* | 15 | -0.117 | 0.677 | 24 | 0.227 | 0.286 |
| *DPB1*31:01* | 12 | -0.870 | 0.000 | 27 | 0.276 | 0.164 |
| *DPB1*32:01* | 12 | 0.091 | 0.779 | 25 | 0.112 | 0.594 |
| *DPB1*33:01* | 13 | -0.382 | 0.198 | 27 | -0.185 | 0.357 |
| *DPB1*34:01* | 14 | 0.054 | 0.853 | 23 | 0.300 | 0.165 |
| *DPB1*35:01* | 13 | -0.651 | 0.016 | 22 | 0.181 | 0.419 |
| *DPB1*36:01* | 10 | -0.623 | 0.054 | 20 | 0.129 | 0.588 |
| *DPB1*37:01* | 10 | -0.418 | 0.230 | 16 | 0.012 | 0.966 |
| *DPB1*38:01* | 9 | -0.596 | 0.090 | 15 | 0.242 | 0.384 |
| *DPB1*39:01* | 15 | 0.264 | 0.343 | 25 | 0.185 | 0.377 |
| *DPB1*40:01* | 14 | -0.016 | 0.956 | 23 | 0.132 | 0.549 |
| *DPB1*41:01* | 8 | -0.596 | 0.119 | 14 | -0.157 | 0.593 |
| *DPB1*44:01* |  |  |  | 15 | -0.410 | 0.129 |
| *DPB1*45:01* | 11 | -0.751 | 0.008 | 18 | 0.047 | 0.853 |
| *DPB1*46:01* | 12 | -0.402 | 0.195 | 20 | 0.118 | 0.622 |
| *DPB1*47:01* | 10 | -0.595 | 0.069 | 17 | 0.631 | 0.007 |
| *DPB1*48:01* | 11 | -0.481 | 0.134 | 20 | -0.297 | 0.203 |
| *DPB1*49:01* | 11 | -0.184 | 0.588 | 22 | 0.308 | 0.163 |
| *DPB1*50:01* | 11 | -0.404 | 0.218 | 18 | 0.053 | 0.836 |
| *DPB1*51:01* | 13 | -0.198 | 0.517 | 27 | -0.140 | 0.485 |
| *DPB1*52:01* | 10 | -0.740 | 0.014 | 17 | -0.438 | 0.079 |
| *DPB1*55:01* | 13 | -0.101 | 0.742 | 21 | 0.140 | 0.545 |
| *DPB1*56:01* | 9 | -0.596 | 0.090 | 15 | -0.181 | 0.519 |
| *DPB1*57:01* | 9 | -0.596 | 0.090 | 16 | -0.140 | 0.604 |
| *DPB1*59:01* | 7 | -0.596 | 0.158 | 13 | 0.253 | 0.404 |
| *DPB1*60:01* | 9 | 0.256 | 0.506 | 15 | 0.351 | 0.200 |
| *DPB1*62:01* | 9 | 0.056 | 0.886 | 16 | 0.457 | 0.075 |
| *DPB1*63:01* | 7 | -0.596 | 0.158 | 13 | 0.253 | 0.404 |
| *DPB1*65:01* | 9 | -0.347 | 0.360 | 17 | -0.196 | 0.452 |
| *DPB1*66:01* | 8 | 0.280 | 0.501 | 14 | -0.230 | 0.429 |
| *DPB1*67:01* |  |  |  | 13 | -0.399 | 0.177 |
| *DPB1*68:01* | 9 | -0.323 | 0.397 | 15 | 0.374 | 0.169 |
| *DPB1*69:01* | 7 | -0.596 | 0.158 | 13 | -0.191 | 0.531 |
| *DPB1*70:01* | 7 | -0.596 | 0.158 | 13 | -0.258 | 0.394 |
| *DPB1*71:01* | 7 | -0.596 | 0.158 | 13 | 0.253 | 0.404 |
| *DPB1*72:01* | 8 | -0.596 | 0.119 | 14 | -0.159 | 0.587 |
| *DPB1*73:01* | 7 | -0.596 | 0.158 | 14 | 0.261 | 0.367 |
| *DPB1*75:01* | 8 | -0.148 | 0.726 | 14 | 0.404 | 0.152 |
| *DPB1*76:01* |  |  |  | 5 | 0.176 | 0.776 |
| *DPB1*80:01* |  |  |  | 5 | 0.828 | 0.084 |
| *DQA1*01:01* | 21 | 0.108 | 0.642 | 59 | 0.013 | 0.920 |
| *DQA1*01:02* | 23 | 0.048 | 0.827 | 59 | 0.360 | 0.005 |
| *DQA1*01:03* | 24 | 0.020 | 0.925 | 61 | -0.351 | 0.006 |
| *DQA1*01:04* | 10 | 0.285 | 0.425 | 26 | -0.095 | 0.644 |
| *DQA1*01:05* | 5 | 0.334 | 0.582 | 18 | -0.054 | 0.831 |
| *DQA1*02:01* | 24 | -0.382 | 0.066 | 64 | -0.034 | 0.788 |
| *DQA1*03:01* | 13 | -0.576 | 0.039 | 47 | -0.242 | 0.102 |
| *DQA1*03:02* | 5 | 0.115 | 0.854 | 18 | 0.132 | 0.602 |
| *DQA1*03:03* | |  |  | 13 | -0.053 | 0.864 |
| *DQA1*04:01* | 25 | -0.156 | 0.458 | 63 | 0.069 | 0.593 |
| *DQA1*05:01* | 23 | -0.089 | 0.685 | 60 | -0.027 | 0.836 |
| *DQA1*05:02* | 7 | 0.463 | 0.296 | 15 | -0.137 | 0.627 |
| *DQA1*05:03* | 5 | -0.738 | 0.155 | 11 | -0.051 | 0.881 |
| *DQA1*05:04* | 5 | 0.379 | 0.530 | 10 | 0.441 | 0.202 |
| *DQA1*05:05* | |  |  | 14 | -0.336 | 0.240 |
| *DQA1*06:01* | 17 | -0.136 | 0.602 | 48 | 0.190 | 0.196 |
| *DQA1*06:02* | |  |  | 5 | 0.261 | 0.672 |
| *DQB1*02:01* | 22 | -0.233 | 0.296 | 70 | -0.003 | 0.983 |
| *DQB1*02:02* | 9 | -0.425 | 0.254 | 34 | 0.013 | 0.941 |
| *DQB1*02:03* | 6 | -0.036 | 0.946 | 16 | -0.062 | 0.819 |
| *DQB1*03:01* | 31 | -0.169 | 0.364 | 81 | -0.115 | 0.307 |
| *DQB1*03:02* | 30 | 0.073 | 0.702 | 81 | 0.039 | 0.733 |
| *DQB1*03:03* | 20 | 0.447 | 0.048 | 67 | 0.230 | 0.061 |
| *DQB1*03:04* | 17 | -0.370 | 0.144 | 39 | -0.300 | 0.063 |
| *DQB1*03:05* | 16 | -0.136 | 0.615 | 34 | 0.014 | 0.936 |
| *DQB1*03:06* | 12 | -0.193 | 0.548 | 19 | 0.114 | 0.643 |
| *DQB1*03:09* | |  |  | 7 | 0.560 | 0.191 |
| *DQB1*03:10* | |  |  | 6 | 0.548 | 0.261 |
| *DQB1*03:11* | |  |  | 5 | 0.134 | 0.830 |
| *DQB1*03:12* | |  |  | 5 | -0.375 | 0.534 |
| *DQB1*04:01* | 18 | 0.125 | 0.622 | 52 | -0.121 | 0.394 |
| *DQB1*04:02* | 29 | -0.107 | 0.581 | 78 | 0.159 | 0.164 |
| *DQB1*05:01* | 27 | -0.277 | 0.162 | 76 | -0.125 | 0.281 |
| *DQB1*05:02* | 24 | 0.047 | 0.828 | 74 | 0.152 | 0.196 |
| *DQB1*05:03* | 21 | 0.084 | 0.718 | 67 | -0.054 | 0.665 |
| *DQB1*05:04* | 15 | -0.311 | 0.259 | 31 | 0.246 | 0.183 |
| *DQB1*06:01* | 20 | -0.057 | 0.812 | 66 | -0.164 | 0.188 |
| *DQB1*06:02* | 28 | 0.008 | 0.968 | 79 | 0.029 | 0.798 |
| *DQB1*06:03* | 28 | -0.207 | 0.290 | 76 | -0.089 | 0.446 |
| *DQB1*06:04* | 25 | -0.282 | 0.172 | 68 | -0.076 | 0.537 |
| *DQB1*06:05* | 15 | -0.217 | 0.437 | 41 | 0.129 | 0.421 |
| *DQB1*06:06* | 15 | 0.120 | 0.671 | 27 | 0.069 | 0.733 |
| *DQB1*06:07* | 14 | -0.690 | 0.006 | 26 | -0.527 | 0.006 |
| *DQB1*06:08* | 16 | 0.339 | 0.200 | 27 | -0.038 | 0.850 |
| *DQB1*06:09* | 21 | 0.384 | 0.086 | 43 | 0.289 | 0.060 |
| *DQB1*06:10* | 11 | -0.208 | 0.539 | 19 | 0.224 | 0.356 |
| *DQB1*06:11* | 7 | 0.772 | 0.042 | 11 | 0.439 | 0.177 |
| *DQB1*06:12* | 11 | 0.301 | 0.369 | 18 | -0.049 | 0.848 |
| *DQB1*06:13* | 10 | -0.112 | 0.757 | 17 | 0.240 | 0.353 |
| *DQB1*06:14* | 10 | -0.193 | 0.594 | 19 | -0.084 | 0.734 |
| *DQB1*06:15* | 10 | -0.244 | 0.498 | 18 | 0.348 | 0.157 |
| *DQB1*06:16* | |  |  | 6 | -0.400 | 0.432 |
| *DQB1*06:17* | |  |  | 7 | 0.085 | 0.855 |
| *DQB1*06:19* | |  |  | 5 | 0.329 | 0.588 |
| *DQB1*06:20* | |  |  | 5 | 0.559 | 0.327 |
| *DRB1*01:01* | 26 | -0.430 | 0.028 | 78 | -0.380 | 0.001 |
| *DRB1*01:02* | 21 | -0.316 | 0.162 | 54 | -0.047 | 0.735 |
| *DRB1*01:03* | 18 | -0.522 | 0.026 | 60 | -0.149 | 0.254 |
| *DRB1*01:04* | 7 | -0.167 | 0.721 | 18 | -0.070 | 0.783 |
| *DRB1*01:06* |  |  |  | 11 | 0.036 | 0.915 |
| *DRB1*01:08* |  |  |  | 6 | 0.426 | 0.399 |
| *DRB1*03:01* | 26 | -0.382 | 0.054 |  |  |  |
| *DRB1*03:02* | 20 | -0.127 | 0.593 | 47 | 0.270 | 0.066 |
| *DRB1*03:03* | 15 | 0.346 | 0.206 | 30 | 0.003 | 0.989 |
| *DRB1*03:04* | 11 | -0.363 | 0.273 | 28 | -0.358 | 0.061 |
| *DRB1*03:05* | 11 | 0.371 | 0.261 | 19 | 0.208 | 0.392 |
| *DRB1*03:06* | 8 | -0.207 | 0.622 | 17 | 0.406 | 0.106 |
| *DRB1*03:07* | 10 | 0.509 | 0.133 | 21 | 0.170 | 0.460 |
| *DRB1*03:08* | 7 | -0.678 | 0.094 | 17 | -0.117 | 0.654 |
| *DRB1*03:09* |  |  |  | 17 | -0.310 | 0.226 |
| *DRB1*03:10* |  |  |  | 16 | -0.216 | 0.421 |
| *DRB1*03:11* | 7 | 0.036 | 0.939 | 16 | 0.100 | 0.713 |
| *DRB1*03:12* | 5 | 0.261 | 0.671 | 13 | 0.542 | 0.056 |
| *DRB1*03:15* |  |  |  | 11 | 0.035 | 0.918 |
| *DRB1*03:16* |  |  |  | 9 | 0.548 | 0.127 |
| *DRB1*03:17* |  |  |  | 11 | -0.068 | 0.843 |
| *DRB1*03:21* |  |  |  | 8 | -0.267 | 0.522 |
| *DRB1*04:01* | 20 | -0.324 | 0.163 | 70 | -0.395 | 0.001 |
| *DRB1*04:02* | 18 | -0.547 | 0.019 | 59 | -0.007 | 0.959 |
| *DRB1*04:03* | 25 | -0.236 | 0.255 | 73 | 0.062 | 0.603 |
| *DRB1*04:04* | 25 | -0.256 | 0.218 | 73 | -0.062 | 0.603 |
| *DRB1*04:05* | 24 | -0.071 | 0.740 | 70 | 0.105 | 0.389 |
| *DRB1*04:06* | 18 | -0.459 | 0.055 | 47 | 0.034 | 0.821 |
| *DRB1*04:07* | 20 | 0.167 | 0.481 | 57 | 0.229 | 0.087 |
| *DRB1*04:08* | 19 | -0.331 | 0.166 | 56 | -0.270 | 0.044 |
| *DRB1*04:09* | 9 | -0.491 | 0.179 | 21 | 0.273 | 0.230 |
| *DRB1*04:10* | 16 | -0.035 | 0.897 | 46 | -0.046 | 0.763 |
| *DRB1*04:11* | 16 | -0.166 | 0.539 | 33 | 0.253 | 0.156 |
| *DRB1*04:12* | 9 | -0.153 | 0.693 | 19 | 0.039 | 0.873 |
| *DRB1*04:13* | 9 | -0.297 | 0.437 | 18 | -0.111 | 0.660 |
| *DRB1*04:14* | 9 | 0.060 | 0.878 | 19 | -0.233 | 0.338 |
| *DRB1*04:15* |  |  |  | 21 | -0.196 | 0.394 |
| *DRB1*04:16* |  |  |  | 16 | -0.260 | 0.331 |
| *DRB1*04:17* | 10 | -0.317 | 0.372 | 19 | 0.099 | 0.687 |
| *DRB1*04:18* |  |  |  | 17 | -0.434 | 0.082 |
| *DRB1*04:19* | 9 | -0.291 | 0.448 | 20 | -0.140 | 0.557 |
| *DRB1*04:20* | 9 | 0.124 | 0.751 | 17 | 0.501 | 0.040 |
| *DRB1*04:21* | 9 | -0.142 | 0.715 | 18 | 0.411 | 0.090 |
| *DRB1*04:23* | 8 | 0.066 | 0.876 | 16 | 0.335 | 0.205 |
| *DRB1*04:24* | 9 | 0.016 | 0.967 | 17 | 0.343 | 0.178 |
| *DRB1*04:25* | 9 | 0.017 | 0.966 | 17 | 0.448 | 0.071 |
| *DRB1*04:26* |  |  |  | 15 | -0.371 | 0.173 |
| *DRB1*04:28* | 6 | 0.288 | 0.581 | 14 | 0.489 | 0.076 |
| *DRB1*04:32* | 6 | 0.288 | 0.581 | 14 | 0.291 | 0.314 |
| *DRB1*04:34* |  |  |  | 9 | 0.548 | 0.127 |
| *DRB1*04:36* |  |  |  | 10 | -0.546 | 0.103 |
| *DRB1*04:38* |  |  |  | 9 | 0.548 | 0.127 |
| *DRB1*04:41* |  |  |  | 8 | 0.387 | 0.343 |
| *DRB1*04:42* |  |  |  | 8 | 0.387 | 0.343 |
| *DRB1*07:01* | 28 | -0.438 | 0.020 | 75 | -0.029 | 0.806 |
| *DRB1*07:03* | 6 | 0.288 | 0.581 | 14 | 0.414 | 0.141 |
| *DRB1*07:04* | 6 | 0.829 | 0.041 | 16 | -0.238 | 0.375 |
| *DRB1*07:05* |  |  |  | 8 | 0.387 | 0.343 |
| *DRB1*07:06* |  |  |  | 8 | 0.617 | 0.103 |
| *DRB1*07:07* |  |  |  | 8 | 0.312 | 0.452 |
| *DRB1*08:01* | 16 | -0.493 | 0.052 | 63 | -0.246 | 0.052 |
| *DRB1*08:02* | 21 | -0.122 | 0.599 | 53 | 0.333 | 0.015 |
| *DRB1*08:03* | 13 | 0.392 | 0.185 | 49 | -0.172 | 0.236 |
| *DRB1*08:04* | 17 | 0.349 | 0.170 | 44 | 0.496 | 0.001 |
| *DRB1*08:05* | 9 | 0.060 | 0.878 | 24 | -0.349 | 0.095 |
| *DRB1*08:06* | 12 | -0.301 | 0.341 | 28 | 0.251 | 0.197 |
| *DRB1*08:07* | 10 | -0.002 | 0.995 | 19 | 0.307 | 0.202 |
| *DRB1*08:08* | 11 | 0.225 | 0.505 | 22 | -0.242 | 0.278 |
| *DRB1*08:09* | 9 | -0.031 | 0.936 | 20 | -0.178 | 0.453 |
| *DRB1*08:10* | 10 | 0.235 | 0.513 | 24 | -0.118 | 0.584 |
| *DRB1*08:11* | 9 | 0.442 | 0.234 | 19 | -0.121 | 0.623 |
| *DRB1*08:12* | 8 | 0.066 | 0.876 | 17 | 0.311 | 0.224 |
| *DRB1*08:14* | 8 | 0.066 | 0.876 | 16 | 0.335 | 0.205 |
| *DRB1*08:15* | 8 | 0.066 | 0.876 | 16 | 0.335 | 0.205 |
| *DRB1*08:18* | 9 | 0.301 | 0.432 | 17 | 0.264 | 0.305 |
| *DRB1*08:19* | 7 | 0.036 | 0.939 | 15 | 0.341 | 0.214 |
| *DRB1*09:01* | 14 | -0.079 | 0.788 | 36 | 0.185 | 0.281 |
| *DRB1*09:02* | 6 | 0.610 | 0.198 | 9 | 0.375 | 0.320 |
| *DRB1*10:01* | 31 | 0.156 | 0.402 | 77 | 0.120 | 0.300 |
| *DRB1*11:01* | 25 | 0.361 | 0.076 | 74 | -0.151 | 0.199 |
| *DRB1*11:02* | 24 | 0.333 | 0.112 | 65 | 0.085 | 0.501 |
| *DRB1*11:03* | 12 | -0.326 | 0.301 | 44 | 0.230 | 0.132 |
| *DRB1*11:04* | 21 | -0.402 | 0.071 | 58 | -0.173 | 0.194 |
| *DRB1*11:05* | 10 | 0.307 | 0.388 | 22 | 0.109 | 0.629 |
| *DRB1*11:06* | 13 | -0.113 | 0.713 | 30 | 0.111 | 0.561 |
| *DRB1*11:07* | 11 | 0.609 | 0.047 | 22 | 0.331 | 0.133 |
| *DRB1*11:08* | 10 | 0.244 | 0.497 | 19 | 0.098 | 0.690 |
| *DRB1*11:09* | 10 | 0.452 | 0.190 | 19 | 0.079 | 0.748 |
| *DRB1*11:10* | 8 | 0.033 | 0.939 | 19 | 0.176 | 0.470 |
| *DRB1*11:11* | 10 | 0.201 | 0.578 | 24 | 0.075 | 0.728 |
| *DRB1*11:12* | 8 | 0.338 | 0.413 | 19 | 0.149 | 0.543 |
| *DRB1*11:13* |  |  |  | 21 | -0.257 | 0.262 |
| *DRB1*11:14* | 11 | 0.378 | 0.251 | 21 | 0.244 | 0.286 |
| *DRB1*11:15* | 9 | -0.275 | 0.473 | 19 | -0.112 | 0.647 |
| *DRB1*11:16* | 10 | 0.413 | 0.236 | 19 | 0.335 | 0.161 |
| *DRB1*11:17* | 9 | 0.060 | 0.878 | 18 | 0.181 | 0.472 |
| *DRB1*11:18* |  |  |  | 20 | -0.243 | 0.302 |
| *DRB1*11:19* | 10 | 0.392 | 0.262 | 20 | 0.291 | 0.214 |
| *DRB1*11:21* |  |  |  | 17 | -0.237 | 0.360 |
| *DRB1*11:22* |  |  |  | 18 | -0.240 | 0.337 |
| *DRB1*11:23* | 8 | 0.066 | 0.876 | 16 | 0.335 | 0.205 |
| *DRB1*11:24* |  |  |  | 17 | -0.237 | 0.360 |
| *DRB1*11:25* |  |  |  | 17 | -0.237 | 0.360 |
| *DRB1*11:26* | 8 | 0.066 | 0.876 | 16 | 0.335 | 0.205 |
| *DRB1*11:27* | 10 | -0.157 | 0.666 | 16 | 0.135 | 0.617 |
| *DRB1*11:28* |  |  |  | 18 | -0.251 | 0.315 |
| *DRB1*11:29* | 8 | 0.066 | 0.876 | 17 | 0.265 | 0.305 |
| *DRB1*11:30* | 6 | 0.288 | 0.581 | 15 | 0.335 | 0.222 |
| *DRB1*11:32* | 6 | 0.288 | 0.581 | 13 | 0.500 | 0.082 |
| *DRB1*11:33* |  |  |  | 13 | 0.202 | 0.508 |
| *DRB1*11:37* |  |  |  | 9 | 0.548 | 0.127 |
| *DRB1*11:39* |  |  |  | 10 | 0.454 | 0.187 |
| *DRB1*11:40* |  |  |  | 9 | 0.548 | 0.127 |
| *DRB1*11:43* |  |  |  | 8 | -0.267 | 0.522 |
| *DRB1*12:01* | 24 | 0.450 | 0.027 | 73 | -0.135 | 0.254 |
| *DRB1*12:02* | 18 | 0.095 | 0.707 | 46 | 0.085 | 0.573 |
| *DRB1*12:03* |  |  |  | 5 | 0.544 | 0.343 |
| *DRB1*12:04* | 9 | -0.171 | 0.660 | 17 | 0.431 | 0.084 |
| *DRB1*12:05* | 8 | 0.403 | 0.323 | 16 | 0.434 | 0.093 |
| *DRB1*12:06* |  |  |  | 12 | 0.116 | 0.719 |
| *DRB1*12:08* |  |  |  | 6 | -0.461 | 0.358 |
| *DRB1*13:01* | 28 | -0.150 | 0.445 | 79 | -0.093 | 0.414 |
| *DRB1*13:02* | 27 | -0.267 | 0.178 | 74 | -0.060 | 0.610 |
| *DRB1*13:03* | 21 | 0.346 | 0.124 | 54 | 0.109 | 0.435 |
| *DRB1*13:04* | 15 | 0.530 | 0.042 | 34 | 0.055 | 0.757 |
| *DRB1*13:05* | 12 | -0.093 | 0.774 | 43 | -0.245 | 0.113 |
| *DRB1*13:06* | 10 | 0.392 | 0.262 | 23 | -0.050 | 0.821 |
| *DRB1*13:07* | 10 | 0.289 | 0.418 | 22 | -0.287 | 0.195 |
| *DRB1*13:08* | 10 | 0.600 | 0.067 | 21 | -0.133 | 0.566 |
| *DRB1*13:09* | 10 | 0.452 | 0.190 | 21 | 0.171 | 0.460 |
| *DRB1*13:10* | 11 | 0.505 | 0.113 | 24 | -0.012 | 0.955 |
| *DRB1*13:11* | 9 | 0.577 | 0.104 | 22 | 0.018 | 0.936 |
| *DRB1*13:12* | 12 | 0.302 | 0.340 | 28 | 0.289 | 0.136 |
| *DRB1*13:13* | 10 | 0.239 | 0.506 | 20 | -0.075 | 0.753 |
| *DRB1*13:14* |  |  |  | 18 | -0.654 | 0.003 |
| *DRB1*13:15* | 9 | 0.060 | 0.878 | 21 | -0.162 | 0.482 |
| *DRB1*13:16* |  |  |  | 17 | -0.434 | 0.082 |
| *DRB1*13:17* | 12 | 0.573 | 0.051 | 20 | 0.398 | 0.082 |
| *DRB1*13:18* |  |  |  | 18 | -0.204 | 0.418 |
| *DRB1*13:19* | 9 | 0.060 | 0.878 | 17 | 0.300 | 0.242 |
| *DRB1*13:20* |  |  |  | 18 | 0.051 | 0.840 |
| *DRB1*13:21* |  |  |  | 18 | -0.250 | 0.316 |
| *DRB1*13:22* |  |  |  | 20 | -0.289 | 0.216 |
| *DRB1*13:23* | 9 | 0.442 | 0.234 | 18 | 0.267 | 0.285 |
| *DRB1*13:24* | 8 | -0.144 | 0.733 | 16 | 0.380 | 0.147 |
| *DRB1*13:25* | 9 | 0.442 | 0.234 | 18 | 0.340 | 0.168 |
| *DRB1*13:26* |  |  |  | 17 | -0.217 | 0.402 |
| *DRB1*13:27* | 8 | 0.066 | 0.876 | 17 | -0.098 | 0.708 |
| *DRB1*13:28* | 8 | 0.066 | 0.876 | 16 | 0.335 | 0.205 |
| *DRB1*13:31* | 8 | 0.442 | 0.273 | 16 | 0.280 | 0.294 |
| *DRB1*13:34* |  |  |  | 14 | -0.323 | 0.261 |
| *DRB1*13:36* |  |  |  | 9 | 0.462 | 0.211 |
| *DRB1*13:37* |  |  |  | 9 | -0.287 | 0.454 |
| *DRB1*13:39* |  |  |  | 8 | 0.153 | 0.717 |
| *DRB1*13:40* |  |  |  | 8 | -0.453 | 0.260 |
| *DRB1*13:41* |  |  |  | 9 | -0.287 | 0.454 |
| *DRB1*13:42* |  |  |  | 9 | 0.316 | 0.408 |
| *DRB1*13:50* |  |  |  | 8 | -0.345 | 0.402 |
| *DRB1*14:01* | 23 | 0.036 | 0.871 | 73 | -0.093 | 0.434 |
| *DRB1*14:02* | 21 | -0.048 | 0.835 | 47 | -0.129 | 0.387 |
| *DRB1*14:03* | 12 | 0.176 | 0.583 | 31 | -0.448 | 0.011 |
| *DRB1*14:04* | 16 | -0.184 | 0.494 | 48 | 0.183 | 0.214 |
| *DRB1*14:05* | 16 | 0.295 | 0.268 | 38 | -0.224 | 0.177 |
| *DRB1*14:06* | 13 | -0.200 | 0.513 | 34 | -0.061 | 0.730 |
| *DRB1*14:07* | 15 | 0.462 | 0.083 | 36 | 0.039 | 0.822 |
| *DRB1*14:08* | 15 | 0.354 | 0.195 | 37 | -0.296 | 0.075 |
| *DRB1*14:09* | 11 | 0.380 | 0.249 | 22 | -0.343 | 0.118 |
| *DRB1*14:10* | 10 | 0.061 | 0.867 | 22 | 0.196 | 0.381 |
| *DRB1*14:11* | 9 | 0.060 | 0.878 | 19 | -0.565 | 0.012 |
| *DRB1*14:12* | 11 | 0.445 | 0.170 | 23 | -0.040 | 0.855 |
| *DRB1*14:13* | 10 | 0.058 | 0.873 | 20 | 0.202 | 0.392 |
| *DRB1*14:14* | 9 | 0.060 | 0.878 | 21 | -0.308 | 0.174 |
| *DRB1*14:15* | 10 | 0.039 | 0.914 | 19 | 0.133 | 0.586 |
| *DRB1*14:16* |  |  |  | 19 | -0.302 | 0.208 |
| *DRB1*14:17* | 10 | 0.392 | 0.262 | 18 | 0.351 | 0.153 |
| *DRB1*14:18* | 8 | 0.066 | 0.876 | 17 | 0.231 | 0.372 |
| *DRB1*14:19* | 9 | 0.442 | 0.234 | 17 | 0.262 | 0.309 |
| *DRB1*14:21* | 9 | 0.373 | 0.322 | 18 | 0.369 | 0.132 |
| *DRB1*14:22* | 8 | 0.066 | 0.876 | 17 | 0.208 | 0.423 |
| *DRB1*14:23* |  |  |  | 17 | -0.509 | 0.037 |
| *DRB1*14:24* |  |  |  | 17 | -0.296 | 0.249 |
| *DRB1*14:25* | 8 | 0.066 | 0.876 | 16 | 0.305 | 0.251 |
| *DRB1*14:26* | 6 | 0.082 | 0.877 | 14 | 0.524 | 0.054 |
| *DRB1*14:27* |  |  |  | 17 | -0.399 | 0.113 |
| *DRB1*14:30* | 8 | 0.066 | 0.876 | 16 | 0.335 | 0.205 |
| *DRB1*14:31* |  |  |  | 16 | 0.358 | 0.174 |
| *DRB1*14:33* |  |  |  | 11 | -0.219 | 0.517 |
| *DRB1*14:34* |  |  |  | 9 | 0.548 | 0.127 |
| *DRB1*14:37* |  |  |  | 9 | -0.382 | 0.310 |
| *DRB1*14:43* |  |  |  | 7 | -0.110 | 0.815 |
| *DRB1*14:44* |  |  |  | 7 | 0.387 | 0.391 |
| *DRB1*14:46* |  |  |  | 6 | 0.447 | 0.374 |
| *DRB1*15:01* | 19 | -0.118 | 0.629 | 67 | -0.199 | 0.106 |
| *DRB1*15:02* | 15 | 0.231 | 0.408 | 55 | 0.362 | 0.007 |
| *DRB1*15:03* | 21 | 0.168 | 0.466 | 45 | 0.307 | 0.040 |
| *DRB1*15:04* | 7 | 0.257 | 0.578 | 19 | 0.541 | 0.017 |
| *DRB1*15:05* | 9 | 0.423 | 0.257 | 17 | 0.453 | 0.068 |
| *DRB1*15:06* | 6 | 0.921 | 0.009 | 15 | 0.616 | 0.014 |
| *DRB1*15:07* | 5 | 0.950 | 0.013 | 14 | 0.436 | 0.119 |
| *DRB1*15:09* |  |  |  | 8 | -0.204 | 0.629 |
| *DRB1*15:10* |  |  |  | 7 | -0.759 | 0.048 |
| *DRB1*15:11* |  |  |  | 7 | 0.430 | 0.336 |
| *DRB1*15:12* |  |  |  | 6 | 0.426 | 0.399 |
| *DRB1*16:01* | 14 | -0.184 | 0.528 | 51 | -0.054 | 0.707 |
| *DRB1*16:02* | 26 | -0.588 | 0.002 | 62 | 0.442 | 0.000 |
| *DRB1*16:03* | 9 | 0.060 | 0.878 | 18 | -0.078 | 0.760 |
| *DRB1*16:04* | 10 | -0.138 | 0.704 | 19 | 0.371 | 0.118 |
| *DRB1*16:05* | 10 | 0.414 | 0.234 | 24 | 0.388 | 0.061 |
| *DRB1*16:07* |  |  |  | 17 | -0.356 | 0.161 |
| *DRB1*16:08* | 9 | 0.442 | 0.234 | 17 | 0.336 | 0.187 |
